# From awe to anxiety: investigating art-induced self-transcendence using virtual reality

**DOI:** 10.3389/fpsyg.2026.1753676

**Published:** 2026-04-16

**Authors:** Monica V. Weedman, Kutter Callaway, Daniel M. Shafer, Tyler S. Greenway, Jo-Ann Tsang, Wade C. Rowatt

**Affiliations:** 1 Department of Psychology and Neuroscience, Baylor University, Waco, TX, United States; 2 Fuller Graduate School of Psychology, Pasadena, CA, United States; 3 Department of Film and Digital Media, Baylor University, Waco, TX, United States; 4 Department of Psychology, Calvin University, Grand Rapids, MI, United States

**Keywords:** anxiety, art, awe, self-transcendent emotion, virtual reality

## Abstract

**Introduction:**

Art has been shown to evoke a range of emotions, yet the mechanisms underlying these responses remain underexplored. Across three studies using between- and within-subjects designs, we examined how viewing original photographic art in virtual reality (VR) influences self-transcendent emotions and related affective states.

**Method:**

In Study 1, participants viewed photographs in one of three VR contexts (museum, warehouse, church sanctuary). In Study 2, participants viewed the same photographs in a VR museum context where the photo scale varied (small vs. large scale). In Study 3, online participants viewed video recordings of the VR museum with or without computer-generated avatars.

**Results:**

Contrary to predictions, across all three studies, self-transcendent positive emotions decreased following the virtual art intervention, even after controlling for cybersickness. However, the virtual art intervention also consistently reduced state anxiety.

**Conclusion:**

Additional alternative explanations and implications are discussed for using VR to elicit self-transcendent emotional experiences to art.

## 1 Introduction

Art occasions myriad psychological responses among viewers. For example, some art enthusiasts stumble into an unexpected encounter with a piece of art that changes how they view reality or understand the arts’ meaning. In other cases, some people meander past the same piece of art on autopilot, perhaps motivated more by who they’re with or where they’re going than the present moment. For over 40,000 years, humans have used art to convey symbolic meaning (Aubert et al., 2019), and emotional responses to art have long been discussed in philosophy, art history, and aesthetics. Empirical investigations of aesthetic judgment likewise date back to early psychophysics (e.g., Fechner, 1876) and have been extensively investigated within the field of neuroaesthetics (e.g., Ramachandran and Hirstein, 1999; Zeki, 1999; Zeki and Lamb, 1994).

However, only relatively recently has empirical psychological research moved beyond a primary focus on aesthetic pleasure or beauty to examine the broader emotional and psychological consequences of art engagement (e.g., Hur et al., 2024; Leder and Nadal, 2014), including its capacity to elicit complex, self-transcendent experiences.

A growing body of empirical psychological research suggests that art can evoke powerful self-transcendent emotions –such as awe, gratitude, elevation, and spiritual transcendence—that shift attention beyond the self and foster a sense of connection with humanity (Callaway et al., 2023; Keltner and Haidt, 2003). These emotions capture the “wow” feeling viewers might experience when standing in front of a famous work of art, and have been linked to a range of prosocial behaviors and enhanced wellbeing (see Stellar et al., 2017). Though recent field research suggests that most art museum patrons report experiencing awe during their visit (Luke, 2021), important questions remain about the conditions that enhance or constrain this effect.

Psychological conceptions of awe closely parallel the aesthetic tradition of the sublime, which has been used to describe experiences characterized by vastness, power, and a breakdown of ordinary comprehension (Burke, 1759/1998; Kant, 2017). Kant characterized the sublime as an aesthetic experience to something infinite, whether it be the mathematical sublime (e.g., contemplating the infinite number of stars in the night sky) or the dynamic sublime (e.g., experiencing threatening natural experiences such as a thunderstorm). Burke further theorized that feelings of terror were at the heart of the experience of the sublime, but that this terror was experienced positively due to the perceiver’s distance from actual threat. Contemporary psychological theory and research mirror much of these philosophical components of awe. *Awe* typically occurs in the presence of something vast that requires additional thought to understand or process (see Keltner and Haidt, 2003). Vastness refers to the size or scale of stimuli (e.g., Niagara Falls). Need for accommodation occurs when interpretations or appraisals do not fit with one’s current schemas or understanding. People report experiencing awe in nature, when listening to music, when witnessing exemplary moral or virtuous acts, and in the presence of aesthetic visual designs, such as art (e.g., Al-Kire et al., 2025; Averbach and Monin, 2022; Luke, 2021).

In addition to awe, other self-transcendent emotions, such as elevation and gratitude, may also be elicited by art (Al-Kire et al., 2025). *Elevation*, which involves feeling uplifted or inspired, is thought to be elicited by exemplars of moral excellence (Algoe and Haidt, 2009). *Gratitude* is most often elicited by the perception that one is the beneficiary of a favor or benefit provided intentionally from someone else (McCullough et al., 2002).

Though models of art perception such as “cognitive mastery” (Leder et al., 2004) may hold true for self-transcendent emotions as well as aesthetic evaluation, the theoretical distinctions made between beauty and the sublime (e.g., Burke, 1759/1998; Kant, 2017; Hur et al., 2024) and the cognitively destabilizing nature of self-transcendent emotions (Keltner and Haidt, 2003) likely necessitate additional considerations. According to Pelowski and Akiba’s (2011) model, cognitive mastery—the extent to which the viewer grasps the meaning of the artwork—is just one piece of the puzzle when it comes to understanding transcendent processes. Several other factors, including the viewer’s mindset and expectations, congruency between expected and observed features of the artwork, and one’s ability to adapt to these discrepancies, all shape the aesthetic and self-transcendent emotions experienced (Pelowski and Akiba, 2011).

Consistent with this model and with contemporary constructionist perspectives of emotion, which propose that the experience of emotion is a top-down process driven by interactions between arousal, core affect, and situational construal (Barrett et al., 2007), research suggests that the *context* in which art is viewed may play an important role in the emotions it evokes. For example, art viewed in a museum or gallery has been shown to elicit higher emotional arousal (Brieber et al., 2015; Szubielska and Imbir, 2021), more positive affect (Brieber et al., 2015), less negative affect, and more subjectively significant emotional reactions (Szubielska and Imbir, 2021), than when viewed in a laboratory setting. These context effects are theoretically well-grounded: environmental cues in museum or gallery settings may, even without conscious awareness, activate cognitive scripts, or schemas, that facilitate emotional and behavioral responses (e.g., Keltner and Gross, 1999) appropriate for engaging with art in a way that a laboratory setting does not.

Although schema-congruent contexts, such as viewing art in a museum, have been shown to enhance aesthetic emotions like interest and liking (Locher et al., 1999), their effect on self-transcendent emotions remains largely unexplored. Although self-transcendent emotions are close cousins of aesthetic emotion in the larger family of positive emotions, there are reasons to believe that they may require different elicitors in the art context. Pelowski and Akiba’s (2011) model of art perception suggests that experiences of transcendence arise not from schema confirmation but from schema violation, and Keltner and Haidt’s (2003) theorizing similarly points to “need for accommodation”—or a need to revise current schemas to make sense of the new stimulus—as an essential characteristic of awe-eliciting experiences.

These competing perspectives present a compelling theoretical tension. If self-transcendent emotions function similarly to other positive or aesthetic emotions, then viewing art in schema-congruent settings—such as museums—should enhance their intensity. However, if transcendence depends on a disruption of existing cognitive frameworks (Keltner and Haidt, 2003; Pelowski and Akiba, 2011), then schema-violating contexts may be more effective in eliciting such emotions. Complicating this dichotomy, recent findings suggest that not all forms of schema violation are equally transformative. Taylor and Uchida (2019) propose that the emotional response to schema incongruence depends on the degree of the violation. For example, encountering art in a church may violate expectations of an art exhibit while still fitting within the bounds of culturally acceptable places for art to be displayed, allowing the new experience to be assimilated into existing schema and potentially fostering awe as an expression of this expansion. In contrast, encountering art in a dimly lit, industrial warehouse may violate expectations so severely that it undermines, rather than expands, one’s existing schema for viewing art, shifting the emotional outcome away from awe and toward confusion or discomfort.

In addition to prompting a need for accommodation, vastness—or the perception of something larger than the self—is necessary for arousal to be appraised as awe (Keltner and Haidt, 2003). According to Keltner and Haidt (2003), perceptions of vastness can be evoked by either *physical magnitude*, as one might experience in the presence of a T. Rex skeleton (Keltner and Haidt, 2003) or in places with expansive views, or *conceptual magnitude*, as one might experience in the presence of something particularly intricate, complex, or impressive, that exceeds one’s normal range of experience. Consistent with this view, nearly half of individuals surveyed after visiting an art museum attributed the awe they experienced to the skill of the artists, while another quarter made attributions to the scale of the artwork (Luke, 2021).

Although there is strong theoretical evidence for the role of context and magnitude in the elicitation of self-transcendent emotion, many existing investigations have been limited in their ability to isolate these effects due to methodological constraints. For example, in addition to manipulating the physical context (museum, laboratory) in which art was viewed, many studies also inadvertently manipulated aspects of the art itself by exposing participants in the laboratory conditions to replications of the original art delivered through projected images or computer-simulated versions of the exhibit (e.g., Szubielska and Imbir, 2021).

The present research leverages virtual reality (VR) to overcome these methodological challenges in studying art-induced self-transcendent emotion. VR uses a head-mounted display to immerse the viewer in a computer-generated three-dimensional space that simulates real-life. This technology allows us to directly manipulate the context in which art is viewed and the physical scale on which art is displayed to investigate the effect of art on self-transcendent emotion while controlling for other confounds.

We are not the first to use virtual reality in this way. Building on the growing use of VR as a tool to study emotion more broadly (see Markowitz and Bailenson, 2023), prior studies have demonstrated that VR is particularly effective for eliciting self-transcendent emotions due to its unique ability to target key components of awe (Chirico et al., 2016). For example, the immersive aspect of VR has been shown to increase the perceived vastness of panoramic nature scenes and elicit greater need for accommodation compared to 2D videos of the same stimuli (Chirico et al., 2017). VR is also capable of eliciting self-transcendence by providing novel perspectives that individuals would not otherwise be able to experience, like viewing Earth as a “pale blue dot” from outer space. Relative to a 2D video recording, experiencing a spacewalk in VR amplified both awe and gratitude (Nelson-Coffey et al., 2019). Recent research using a computer-based art gallery intervention suggests that perceived immersion also plays an important role in facilitating emotional responses to art (Alpys et al., 2025), but few studies have used art as stimuli in VR.

Although our primary focus is on the effect of viewing lens-based art in VR on self-transcendent emotions (STEs), we also explore its potential effect on anxiety. Prior work has shown that viewing art online (Trupp et al., 2022) and colorful imagery in VR (Lu and Li, 2025) can reduce anxiety and improve mood, suggesting that alongside increases in STEs, our VR art intervention might also decrease state anxiety. However, theoretical perspectives on STEs and research on VR’s physiological effects complicate this hypothesis. For instance, the need for accommodation considered essential to awe can also produce discomfort or anxiety when new experiences cannot be assimilated into existing schemas (Pelowski and Akiba, 2011; Taylor and Uchida, 2019). Likewise, “cybersickness”—negative physiological symptoms associated with VR such as oculomotor problems, disorientation, and nausea (Caserman et al., 2021; Kennedy et al., 1993)—has been shown to positively correlate with state anxiety (Bouchard et al., 2021) and reduce perceived presence in VR (Riches et al., 2019). Thus, to the extent that the VR art intervention exceeds individuals’ capacity for assimilation with existing experiences of art galleries in real-life, or causes physical discomfort, it may increase anxiety, rather than reduce it, regardless of the intervention’s effect on STEs otherwise (Nelson-Coffey et al., 2019).

### 1.1 The current research

Across three preregistered studies, we investigated the conditions in which art elicits self-transcendent emotions. In Study 1, we tested the effect of context on self-transcendence by randomly assigning participants to view photographic art in one of three virtual reality environments: a museum, a church, or a warehouse. Computer-generated people, not capable of interacting with participants, were included so the participant was not alone in the VR environment. In Study 2, we tested the effect of physical scale on self-transcendence by randomly assigning participants to experience a virtual reality museum in which the photographic art was displayed on a large or small scale. In Study 3, we used video recordings of the museum-like virtual context to test additional parameters potentially affecting STEs, such as the presence of computer-generated people or non-playable characters (NPCs) and the physical symptoms associated with VR (i.e., cybersickness).

All studies were preregistered. All preregistered analyses are reported in this manuscript and in Supplementary material. Data were analyzed using *R* (version 4.3.3; R Core Team, 2024). Where applicable, the Benjamini-Hochberg procedure was implemented to control for inflated false discovery (Benjamini and Hochberg, 1995). Data, materials, analytic code, and preregistrations are available at https://www.osf.io/uyj3h/overview?view_only=e4fb390feb004eda9d8e2ac2123803cd.

## 2 Study 1

The purpose of Study 1 was to investigate the effect of environmental context on art’s ability to elicit self-transcendent emotions like awe, elevation, gratitude, and spiritual transcendence (cf. Al-Kire et al., 2025). Using virtual reality to experimentally manipulate the environment in which a gallery of photographic art was displayed, we predicted that art viewed in contexts consistent with art-viewing schemas (i.e., museum or church sanctuary) would elicit greater self-transcendent emotions than art viewed in contexts causing discrepancies with one’s schema for an art gallery (i.e., warehouse). We also predicted that other schemas associated with specific contexts would affect the type of self-transcendence experienced. Specifically, we expected art displayed in the church environment to elicit greater spiritual transcendence relative to other self-transcendent emotions, whereas art displayed in secular contexts (i.e., museum, warehouse) would demonstrate the reverse. Although not preregistered, we also explored the effect of context on state anxiety.

### 2.1 Method

#### 2.1.1 Participants and procedure

A priori power analysis in G*Power indicated that a sample size of 380 participants would provide adequate power (0.80) to detect a small effect between conditions when α = 0.05. To account for exclusions due to inattentiveness and incomplete responses, we preregistered that we would oversample by 15%, resulting in a total sample size of 438 participants. However, with IRB approval, we deviated from this preregistered sample size to recruit a total of 483 participants, due to the high frequency of technical difficulties encountered with the VR software early in data collection. This decision was made prior to viewing any study data.

Participants were recruited from our university’s SONA undergraduate subject pool and were compensated with course credit. After exclusions due to incompleteness, a total of 464 participants were included in analyses. See Table 1 for a description of the sample (i.e., gender, age, race-ethnicity).

**TABLE 1:** Study 1 Participant demographics by VR condition.

Demographic	Church (*n* = 148)		Museum (*n* = 165)		Warehouse (*n* = 151)		Full sample (*N* = 464)	
	*N*	*%*	*N*	*%*	*N*	*%*	*N*	*%*
Gender								
Women	117	79	133	81	115	76	365	79
Men	29	20	30	18	34	23	93	20
Other	2	1	1	< 1	1	<1	5	1
Race								
White	81	55	85	52	92	61	258	56
Hispanic/ Latino	17	11	30	18	22	15	69	15
Asian	18	13	22	13	13	9	54	12
Black	12	8	12	7	10	7	34	7
Multiracial	11	7	14	8	7	5	31	7
Other	8	5	1	< 1	6	4	15	3
Religion								
Christian	128	86	139	84	128	85	395	85
None	4	3	8	5	3	2	15	3
Agnostic	3	2	11	7	7	5	21	5
Hindu	5	3	4	2	2	1	11	2
Atheist	2	1	2	1	5	3	9	2
Age	19.14 (1.32)		19.59 (2.42)		19.20 (1.55)		19.32 (1.86)	

Church, Museum, and Warehouse refer to the VR context experienced by the participant.

##### 2.1.1.1 Pre-test

Upon arrival, participants were directed to a 10’ × 10’ room equipped with a standard desktop computer and VR equipment (describe below). After providing informed consent, participants responded to pre-test measures of their current self-transcendent emotions (elevation, gratitude), negative emotions, and spiritual transcendence, in an online survey.

##### 2.1.1.2 Virtual reality

**FIGURE 1:**
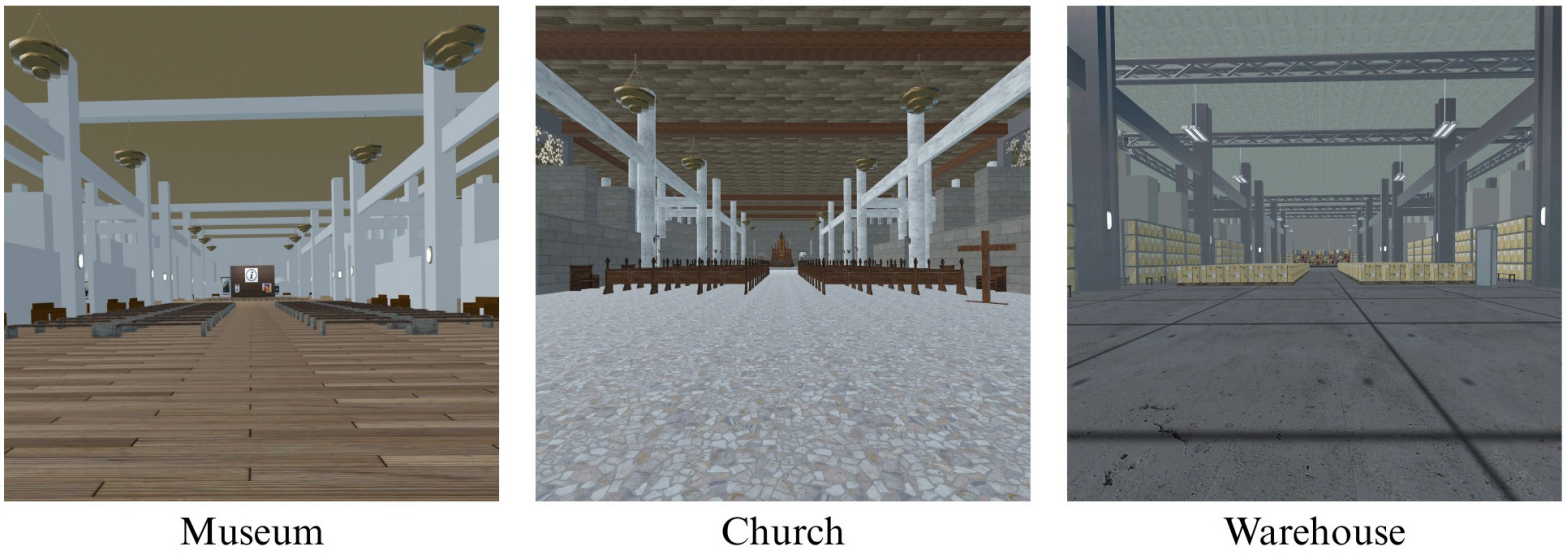
Study 1 virtual reality environments.

Following completion of pre-test measures, participants were randomly assigned via Qualtrics randomization to one of three conditions determining the virtual environment in which participants would view an art display: (1) *Museum*, (2) *Church*, or (3) *Warehouse* (see Figure 1). All three VR environments were developed by an experienced software developer (Michael Villano, University of Notre Dame), based on the same physical dimensions but designed to resemble a church sanctuary, museum, or warehouse. Professionally produced photographic art depicting a wide range of content (e.g., nature, people, buildings, abstract) and found to elicit self-transcendent emotions in prior research (Al-Kire et al., 2025) was displayed on the walls of the virtual space in randomized order. In an attempt to increase realism, computer-generated, non-interactive characters or NPCs, were included.

Participants were fitted with a Vive Pro 2 VR head mounted display. They used a Vive wireless motion-sensing controller with trackpad to navigate the virtual space. Participants were told that they would have 20 min to explore the space but could end the virtual reality experience at any time by notifying the researcher. Participants were asked to view each piece of art at least once.

##### 2.1.1.3 Post-test

After completing the virtual reality experience, participants returned to the desktop computer to complete post-test measures which included self-transcendent (awe, elevation, gratitude) and negative emotions experienced during the virtual art gallery, current spiritual transcendence and cybersickness, and demographics. After completing the post-test survey, participants were debriefed.

#### 2.1.2 Measures

##### 2.1.2.1 Self-transcendent emotion

The self-transcendent emotions of awe, elevation, and gratitude were assessed as states.

*State awe* was assessed after the virtual reality experience (post-test) by Yaden et al.’s (2019) 30-item Awe-Experience Scale, containing six dimensions of awe: altered time perception (5 items; e.g., “I noticed time slowing”), self-diminishment (5 items; e.g., “I felt that my sense of self was diminished”), physical sensation (5 items; e.g., “I had goosebumps”), vastness (5 items; e.g., “I felt that I was in the presence of something grand”), connectedness (5 items; e.g., “I had the sense of being connected to everything”), and need for accommodation (5 items; e.g., “I felt challenged to mentally process what I was experiencing”). Asked to reflect on their virtual art viewing experience, participants indicated the extent to which they agreed or disagreed with each statement on a 7-point Likert-type scale (1 = *Strongly disagree*, 7 = *Strongly agree*).

*State elevation* (moved, uplifted, inspired, warm, optimistic) and *state gratitude* (grateful, thankful, appreciative) were assessed before (pre-test) and after (post-test) the virtual reality experience using Algoe and Haidt’s (2009) scale of other-praising emotions, a measure similar to the widely used modified differential emotions scale (mDES; Fredrickson, 2001). Participants indicated the extent to which they felt each emotion on a 5-point Likert-type scale (1 = *Very slightly*, 5 = *Extremely*).

##### 2.1.2.2 Spiritual transcendence

Spiritual transcendence was assessed before (pre-test) and after (post-test) the virtual reality experience by Abernethy and Kim’s (2018) 8-item Spiritual Transcendence Index. Participants indicated the extent to which they agreed or disagreed with statements like, “My spirituality gives me a feeling of fulfillment,” on a 6-point Likert-type scale (1 = *strongly disagree*, 6 = *strongly agree*).

##### 2.1.2.3 State anxiety

State anxiety was assessed before (pre-test) and after (post-test) the virtual reality experience using a 4-item subscale from the Discrete Emotions Questionnaire (Harmon-Jones et al., 2016). Participants indicated the extent to which they currently felt dread, anxiety, nervous, and worry on a 7-point scale (1 = *not at all*, 7 = *an extreme amount*).

##### 2.1.2.4 Cybersickness

Cybersickness was measured after the virtual reality experience (post-test) using Kennedy et al.’s (1993) Simulator Sickness Questionnaire. Participants rated the extent to which they were affected by symptoms such as “eye strain” and “vertigo” on a 4-point Likert type scale (1 = *None*, 4 = *Severe*).

##### 2.1.2.5 Experience with virtual reality

Prior experience with virtual reality was assessed by two items: “How many times have you worn a virtual reality (VR) headset?” and “In the average week, how many hours do you spend wearing a virtual reality (VR) headset?.” Participants responded to each question on a scale from *0* to *6 or more.*

##### 2.1.2.6 Experience with art

Prior experience with art was assessed by six items from the Art Experience Questionnaire (Chatterjee et al., 2010), such as “In the average week, how many hours do you spend each week looking at visual art?” Participants responded to each question on a scale from *0* to *6 or more*.

##### 2.1.2.7 Demographics

Participants responded to demographic measures, including age, gender, race, and religious affiliation.

### 2.2 Results and discussion

#### 2.2.1 Preliminary analyses

All variables demonstrated good reliability (α = 0.74–98). See Table 2 for descriptive statistics. Conditions did not differ in their prior experience with VR, *F*(2, 461) = 2.02, *p* = 0.13, or art, *F*(2, 461) = 0.93, *p* = 0.39, with all participants reporting having had little experience with either.

**TABLE 2:** Descriptive statistics for study 1 variables by VR condition.

Variable	Church (*n* = 148) M (SD)	Museum (*n* = 165) M (SD)	Warehouse (*n* = 151) M (SD)	All (*N* = 464) M (SD)	α
Pre-VR					
Elevation	2.95 (0.87)	2.91 (0.90)	2.95 (0.98)	2.94 (0.91)	0.87
Gratitude	3.77 (0.92)	3.80 (0.96)	3.88 (0.92)	3.82 (0.93)	0.92
Spiritual transcendence	4.61 (1.28)	4.44 (1.39)	4.45 (1.43)	4.50 (1.37)	0.97
Anxiety	2.56 (1.34)	2.59 (1.39)	2.52 (1.26)	2.56 (1.33)	0.86
*Post-VR*					
Awe	3.81 (0.86)	3.60 (0.97)	3.58 (0.96)	3.66 (0.93)	0.92
Altered time	5.01 (1.09)	4.75 (1.34)	4.62 (1.49)	4.79 (1.32)	0.89
Self-diminishment	3.97 (1.51)	3.58 (1.66)	3.68 (1.66)	3.74 (1.62)	0.91
Connectedness	2.99 (1.38)	3.03 (1.47)	2.71 (1.49)	2.91 (1.45)	0.92
Vastness	3.74 (1.45)	3.46 (1.62)	3.26 (1.46)	3.49 (1.52)	0.90
Physical sensation	2.59 (1.18)	2.50 (1.23)	2.57 (1.27)	2.55 (1.23)	0.82
Need for accommodation	4.55 (1.43)	4.25 (1.49)	4.63 (1.29)	4.47 (1.41)	0.85
Elevation	2.81 (0.99)	2.88 (1.09)	2.71 (0.98)	2.80 (1.05)	0.91
Gratitude	3.48 (1.10)	3.50 (1.10)	3.21 (1.17)	3.40 (1.13)	0.95
Spiritual transcendence	4.68 (1.35)	4.46 (1.47)	4.46 (1.49)	4.53 (1.44)	0.98
Anxiety	1.86 (0.98)	1.92 (1.08)	2.11 (1.08)	1.96 (1.05)	0.82
Cybersickness	1.67 (0.50)	1.67 (0.53)	1.64 (0.49)	1.66 (0.51)	0.90
Experience with VR	1.02 (0.96)	1.17 (0.95)	1.24 (1.01)	1.15 (0.98)	
Experience with art	0.67 (0.81)	0.60 (0.67)	0.55 (0.68)	0.61 (0.72)	0.74

Participants spent an average of 13.03 min (*SD* = 5.08) exploring the virtual environment. Time spent in the virtual environment did not significantly differ between sanctuary (*M* = 13.17, *SD* = 5.08), museum (*M* = 13.27, *SD* = 5.01), and warehouse conditions (*M* = 12.64, *SD* = 5.17), *F*(2, 408) = 0.62, *R*^2^ = 0.00, *p* = 0.54. All virtual environments were perceived as moderately realistic (*M* = 3.12, *SD* = 1.57), with slightly more realistic art (*M* = 4.94, *SD* = 1.22), and mostly unrealistic computer-generated characters (simulated people) (*M* = 1.66, *SD* = 1.57). Conditions did not differ in these ratings.

VR context conditions did not significantly differ in reported cybersickness, *F*(2, 461) = 0.16, *p* = 0.85. Because cybersickness was only assessed at Time 2 (after viewing art in VR), we conducted exploratory analyses on the strength of association between self-indicated cybersickness and emotional states (i.e., awe, awe facets, elevation, and spiritual transcendence) across conditions. As shown in Table 3, cybersickness correlated positively with anxiety, but also awe-self-diminishment and awe-need-for-accommodation. In contrast, cybersickness correlated negatively with awe-connectedness and elevation. Although it seems intuitive to predict an inverse relationship between cybersickness and positive emotions, the fact that awe subscales correlated in different directions with cybersickness makes it difficult to justify such a prediction (at least with regard to the total scale score for awe). As such, we attempted to replicate this pattern in Study 2. Due to the associations between cybersickness and our dependent variables, we chose to control for cybersickness in the analyses below, deviating from our preregistered analysis plan. See Supplementary material for results of analyses without controlling for cybersickness.

**TABLE 3:** Correlations between study 1 post-test variables.

Variable	1.	2.	3.	4.	5.	6.	7.	8.	9.	10.	11.	12.	13.	14.
1. Cybersickness	1	1	1	1	1	1	1	1	1	1	1	1	1	1
2. VR experience	–0.09													
3. Art experience	0.07	0.06												
4. Awe	0.08	–0.11*	–0.02											
5. Altered time-perception	0.05	–0.04	–0.04	0.64***										
6. Self-diminishment	0.22***	–0.07	0.03	0.62***	0.38***									
7. Connectedness	–0.13**	–0.04	–0.00	0.63***	0.25***	0.11*								
8. Vastness	–0.08	–0.03	–0.00	0.79***	0.34***	0.30***	0.66***							
9. Physical sensation	0.06	–0.10*	0.01	0.66***	0.25***	0.29***	0.36***	0.49***						
10. Need for accommodation	0.17***	–0.13**	–0.07	0.58***	0.34***	0.29***	0.09	0.28***	0.30***					
11. Elevation	–0.27***	–0.06	0.04	0.38***	0.16***	0.08	0.45***	0.42***	0.30***	0.09				
12. Gratitude	–0.25***	–0.08	0.03	0.24***	0.13**	0.01	0.31***	0.30***	0.15**	0.04	0.73***			
13. Spiritual Transcendence	–0.03	–0.09	–0.09	0.22***	0.16***	0.19***	0.11*	0.20***	0.02	0.18***	0.25***	0.27***		
14. Anxiety	0.41***	–0.03	0.04	0.11*	0.11*	0.11*	–0.09	–0.04	0.11*	0.22***	–0.19***	–0.17***	–0.06	

*p < 0.05, **p < 0.01, ***p < 0.001.

#### 2.2.2 Self-transcendent emotions

To test for effects of VR context on the self-transcendent emotion of awe, a series of 3 × 1 ANCOVAs were performed with condition (church, museum, warehouse) as the independent variable, cybersickness as an exploratory covariate, and each subscale of awe as the dependent variable, assessed in separate models. Cybersickness was a significant covariate in models predicting self-diminishment, *F*(1, 460) = 23.76, η_*p*_^2^ = 0.05, *p* < 0.001, connectedness, *F*(1, 460) = 7.88, η_*p*_^2^ = 0.02, *p* < 0.01, and need for accommodation, *F*(1, 460) = 13.74, η_*p*_^2^ = 0.03, *p* < 0.001.

**FIGURE 2:**
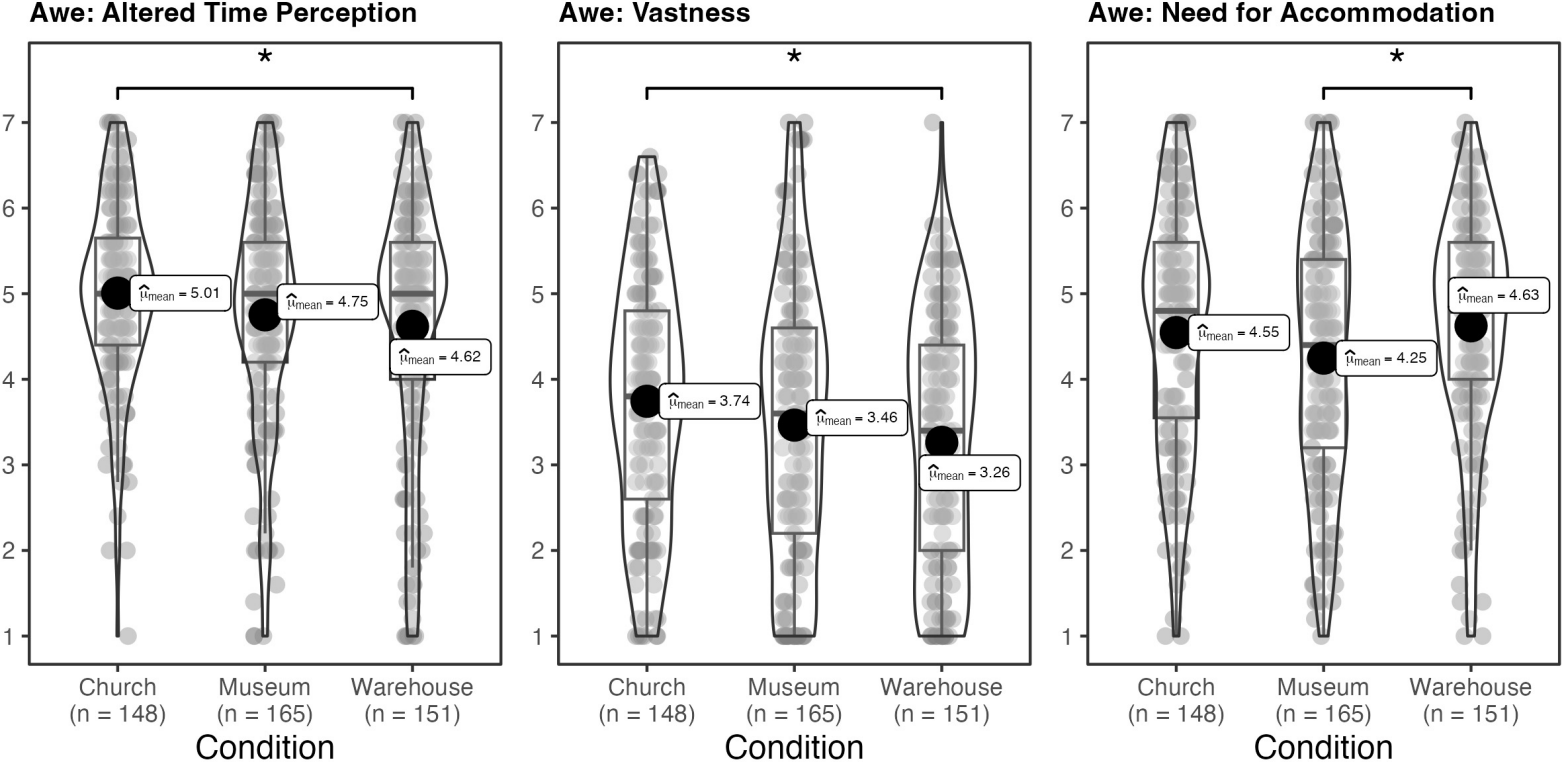
Study 1: effect of condition on dimensions of awe. *p < 0.05.

Although conditions did not significantly differ in awe when assessed by the full scale, *F*(2, 460) = 2.79, η_*p*_^2^ = 0.01, *p*_*adj.*_ = 0.06, exploratory analyses indicated that conditions did significantly differ on subscales of awe regarding altered time perception, *F*(2, 460) = 3.28, η_*p*_^2^ = 0.01, *p*_*adj.*_ < 0.05, vastness, *F*(2, 460) = 3.90, η_*p*_^2^ = 0.02, *p*_*adj.*_ < 0.05, and need for accommodation, *F*(2, 460) = 3.46, η_*p*_^2^ = 0.01, *p*_*adj.*_ < 0.05 (see Figure 2). As shown in Figure 2, participants in the warehouse environment reported less altered time perception [*t*(460) = -2.53, *d* = 0.29, CI (0.06, 0.52), *p*_*adj*_. < 0.05] and vastness [*t*(460) = -2.78, *d* = 0.32, CI (0.09, 0.55), *p*_*adj*_. < 0.05] than those in the church environment and reported greater need for accommodation [*t*(460) = 2.50, *d* = 0.28, CI (0.06, 0.50), *p*_*adj*_. < 0.05] than those in the museum environment. Conditions did not significantly differ in self-diminishment [*F*(2, 460) = 2.50, η_*p*_^2^ = 0.01, *p*_*adj.*_ = 0.08], connectedness [*F*(2, 460) = 2.49, η_*p*_^2^ = 0.01, *p*_*adj.*_ = 0.08], or physical sensation [*F*(2, 460) = 0.26, η_*p*_^2^ = 0.00, *p*_*adj.*_ = 0.77].

To test for effects of art in VR on self-transcendent emotions of elevation and gratitude, a series of 3 × 2 ANCOVAs were performed with pre- and post-test measures of each STE as the within-subjects factor (time), VR condition (church, museum, warehouse) as the between-subjects factor, and cybersickness as an exploratory covariate. Cybersickness was a significant covariate in models predicting elevation, *F*(1, 458) = 19.03, *p_*adj*_.* < 0.001, and gratitude, *F*(1, 458) = 15.32, *p* < 0.001.

**FIGURE 3:**
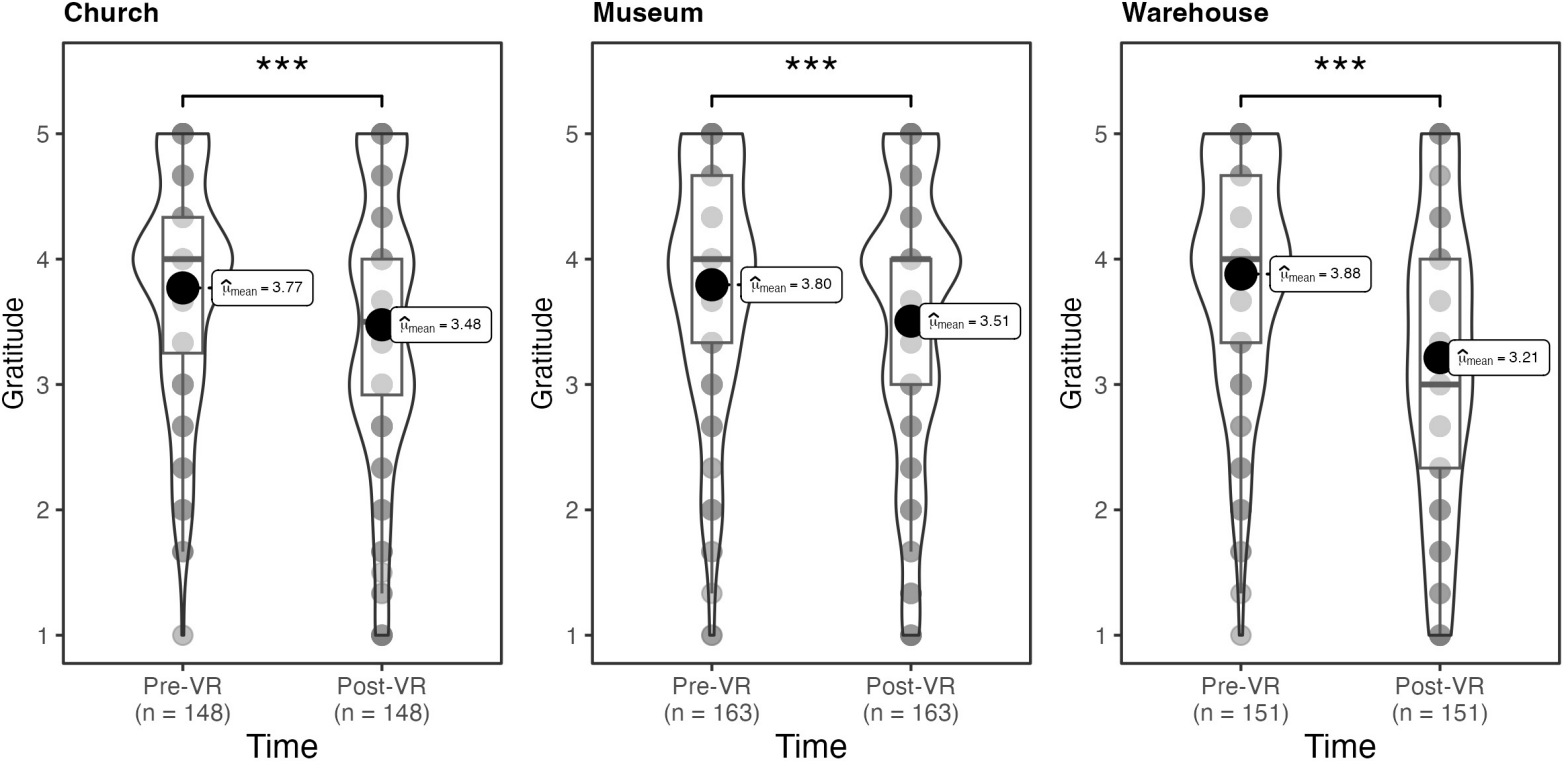
Study 1: effect of condition on gratitude. ***p < 0.001.

Contrary to our hypothesis that viewing art in VR would increase self-transcendent emotions, state elevation, *F*(1, 458) = 20.07, η_*G*_^2^ = 0.01, *p_*adj.*_* < 0.001, and state gratitude, *F*(1, 458) = 8.92, η_*G*_^2^ = 0.004, *p_*adj.*_* < 0.01, significantly decreased from pre-test to post-test. Whereas the presentational context of photographic art did not significantly influence state elevation, *F*(2, 458) = 2.94, η_*G*_^2^ = 0.003, *p_*adj.*_* = 0.07, a significant interaction between context and time was detected for state gratitude, *F*(2, 458) = 9.71, η_*G*_^2^ = 0.01, *p*_*adj.*_ < 0.001. As shown in Figure 3, although participants in the church [*t*(459) = 3.85, *d* = 0.45, *p*_*adj*_. < 0.001] and museum [*t*(459) = 4.03, *d* = 0.45, *p*_*adj*_. < 0.001] conditions also reported significant drops in gratitude from pre-test to post-test, this decrease was most dramatic among participants in the warehouse condition, *t*(459) = 8.82, *d* = 1.01, *p*_*adj*_. < 0.001.

#### 2.2.3 Spiritual transcendence

We conducted exploratory tests for effects of art in VR on spiritual transcendence using a 3 × 2 ANCOVA with pre- and post-test measures of spiritual transcendence as the within-subjects factor (time), VR condition (church, museum, warehouse) as the between-subjects factor, and cybersickness as an exploratory covariate. Cybersickness was not a significant covariate, *F*(1, 457) = 0.35, η_*G*_^2^ = 0.00, *p*_*adj.*_ = 0.56.

We did not find support for our exploratory hypothesis that viewing art in the virtual church environment would increase spiritual transcendence relative to the other presentational contexts. Instead, spiritual transcendence remained stable from pre-test to post-test, *F*(1, 457) = 2.14, η_*G*_^2^ = 0.00, *p*_*adj.*_ = 0.44, and this null effect was consistent across conditions, *F*(2, 457) = 1.37, η_*G*_^2^ = 0.00, *p*_*adj.*_ = 0.44.

#### 2.2.4 Anxiety

We conducted exploratory analyses on the effect of VR context on state anxiety using a 3 × 2 ANCOVA with pre- and post- test measures of state anxiety as the within-subjects factor (time), VR condition (church, museum, warehouse) as the between-subjects factor, and cybersickness as a covariate. Cybersickness was a significant covariate, *F*(1, 458) = 63.35, *p_*adj*_.* < 0.001, η_*G*_^2^ = 0.09.

**FIGURE 4:**
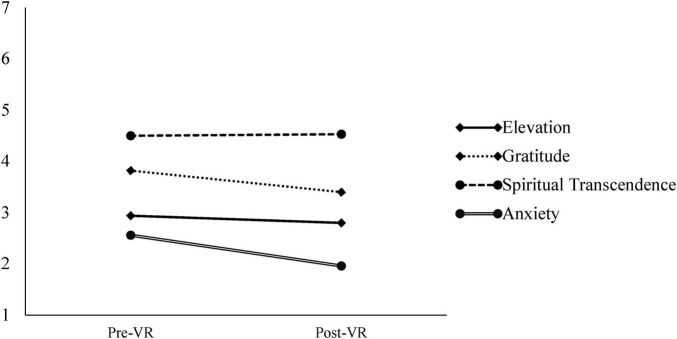
Study 1: dependent variables over time. Means are shown to illustrate change in each variable from pre-test (pre-VR) to post-test (post-VR). Variables are assessed on different scales and should not be directly compared; Elevation and gratitude = 1–5; Spiritual transcendence = 1–6; Anxiety = 1–7.

We observed a significant main effect of time (pre- to post-VR) on state anxiety, *F*(1, 458) = 30.89, η_*G*_^2^ = 0.02, *p*_*adj.*_ < 0.001. As shown in Figure 4, state anxiety significantly decreased from pre-test to post-test, following a similar pattern to many of the self-transcendent emotions. Non-significant interactions between condition and time, *F*(2, 458) = 2.34, η_*G*_^2^ = 0.00, *p*_*adj.*_ = 0.10, suggest that this effect did not differ by condition.

## 3 Study 2

In Study 1, we experimentally manipulated the virtual environment (i.e., church, museum, or warehouse) in which the art gallery was displayed to investigate the role of context on self-transcendent emotions. Contrary to our hypotheses, we did not find evidence that self-transcendent emotions increased after viewing art in virtual reality, nor did we find consistent context effects on self-transcendent emotions. One possibility is that the art stimuli in Study 1 were not perceived to be vast. In Study 2, we use VR to manipulate perceived vastness of the art stimuli (i.e., photograph size) in the museum context. We predicted that art presented on a large scale would elicit greater self-transcendence than art presented on a small scale (Hur et al., 2024).

### 3.1 Methods

#### 3.1.1 Participants and procedure

A priori power analyses in G*Power indicated that a sample size of 200 participants would provide adequate power (0.80) to detect a small within-between interaction effect and a sample size of 264 participants would provide adequate power (0.80) to detect a small between-subjects effect, when α = 0.05. To account for exclusions due to inattentiveness and incomplete responses, we preregistered that we would oversample by 25%, resulting in a total sample of 250 participants. However, with IRB approval, we deviated from this preregistered sample size to recruit a total of 277 participants to provide greater power to detect between-subjects effects. This decision was made prior to viewing any study data.

Participants were recruited from our university’s SONA undergraduate subject pool (*n* = 239) and via flyers posted off-campus (*n* = 38). Individuals who participated in Study 1 were excluded from participating in Study 2. Undergraduate student participants recruited via SONA were compensated with course credit. Non-student participants recruited via flyer were compensated with a $20.00 gift card. After exclusions due to incompleteness, a total of 263 participants were included in analyses. See Table 4 for a description of the sample (i.e., gender, age, race-ethnicity).

**TABLE 4:** Study 2 participant demographics by VR condition.

Demographic	Small (*n* = 128)		Large (*n* = 135)		Full sample (*n* = 263)	
	*N*	*%*	*N*	*%*	*N*	*%*
Gender						
Women	82	64	89	66	171	65
Men	42	33	45	33	88	34
Other	1	< 1	1	<1	2	< 1
Race						
White	67	52	72	53	139	53
Latino	17	13	25	19	42	16
Asian	21	16	19	14	40	15
Black	8	6	11	8	19	7
Multiracial	11	9	6	4	17	6
Other	3	2	2	1	5	2
Religion						
Christian	100	78	106	79	206	78
Hindu	6	5	7	5	13	5
Muslim	2	2	2	1	4	2
Atheist	0	0	2	1	2	< 1
Agnostic	3	2	4	3	7	3
Other	17	13	14	10	31	12
Age	19.69 (1.72)		20.34 (3.78)		20.02 (2.98)	

Small and Large refer to the VR context experienced by the participant.

##### 3.1.1.1 Pre-test

Upon arrival, participants were directed to a 10’ × 10’ room equipped with a standard desktop computer and VR equipment (described below). After providing informed consent, participants completed pre-test measures of their current self-transcendent and negative emotions in an online survey.

##### 3.1.1.2 Virtual reality

**FIGURE 5:**
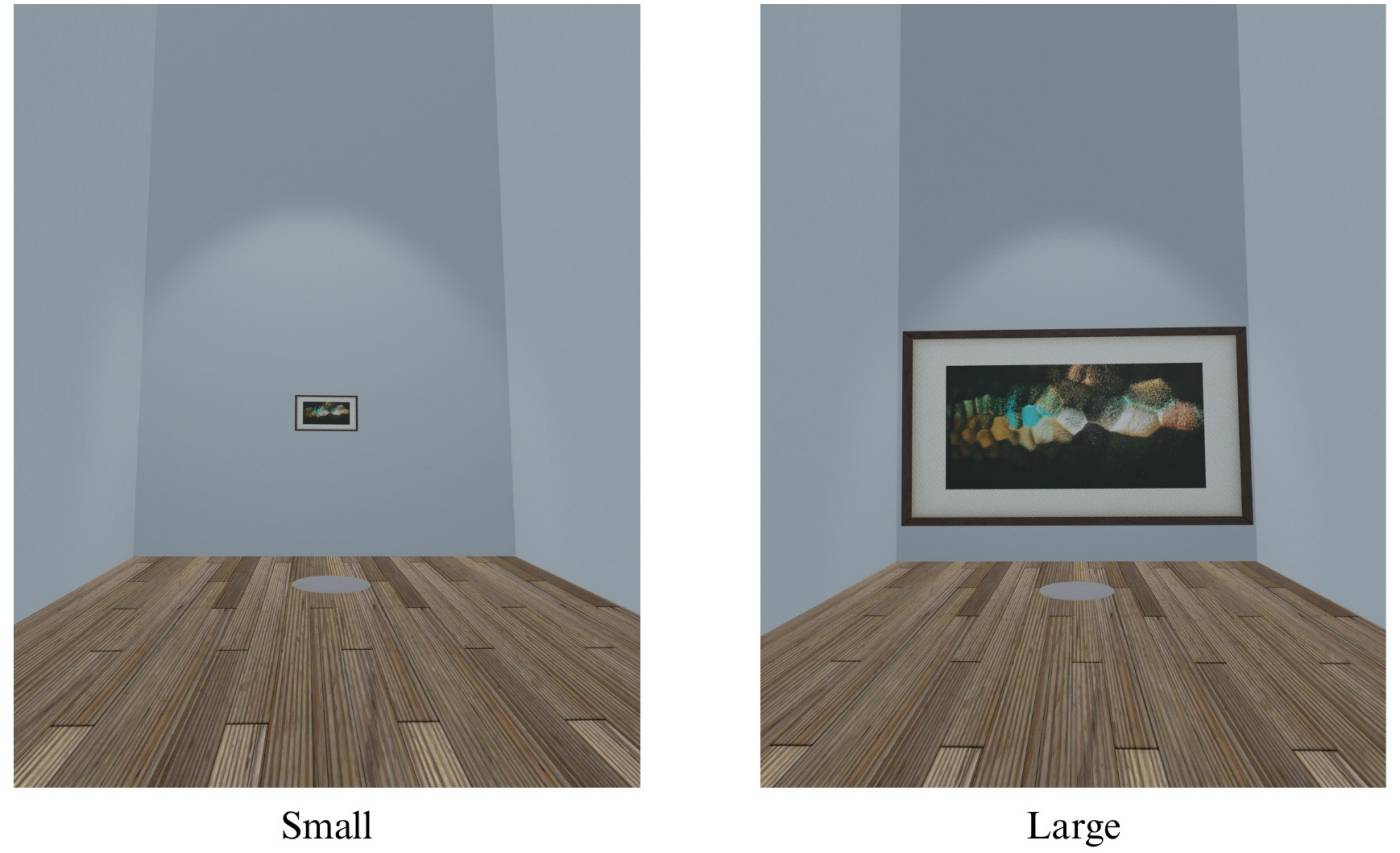
Study 2 virtual reality environments.

Following completion of pre-test measures, participants were randomly assigned via Qualtrics randomization to one of two conditions determining the size of the art displayed in the virtual environment: (1) *Small* or (2) *Large* (see Figure 5). The art in the *Large* condition was sized to cover the entire display wall and was five times larger than the art in the *Small* condition. Both conditions used the same photographic art stimuli and were set in the museum-like virtual environment from Study 1.

Participants were fitted with a Vive Pro 2 VR head mounted display. They used a Vive wireless motion-sensing controller with trackpad to navigate the virtual space. Participants were told that they would have 20 min to explore the space but could end the virtual reality experience at any time by notifying the researcher. Participants were asked to view each piece of art at least once.

##### 3.1.1.3 Post-test

After completing the VR experience, participants returned to the desktop computer to complete post-test self-report measures reflecting on their virtual art gallery experience.

#### 3.1.2 Measures

##### 3.1.2.1 Self-transcendent emotions and spiritual transcendence

Awe, elevation and spiritual transcendence were measured and assessed as described in Study 1. Unlike in Study 1, state awe was assessed before (pre-test) the virtual reality experience as well.

##### 3.1.2.2 State anxiety

Anxiety was assessed as described in Study 1.

##### 3.1.2.3 Control variables

Cybersickness, experience with VR, and experience with art, were measured and assessed as described in Study 1.

### 3.2 Results and discussion

#### 3.2.1 Preliminary analyses

All variables demonstrated acceptable reliability (α = 0.76–98). See Table 5 for descriptive statistics. As in Study 1, conditions did not differ in their prior experience with VR, *F*(1, 260) = 0.73, *p* = 0.39, or art, *F*(1, 260) = 0.13, *p* = 0.71, with all participants reporting having had little experience with either.

**TABLE 5:** Descriptive statistics for study 2 variables by VR condition.

Variable	Small (*n* = 128)		Large (*n* = 135)		Full Sample (*n* = 263)		α (pre/post)
	Pre-VR	Post-VR	Pre-VR	Post-VR	Pre-VR	Post-VR	
Awe	3.54 (0.92)	3.45 (1.03)	3.55 (1.07)	3.78 (1.08)	3.55 (1.00)	3.62 (1.07)	0.94/0.94
Altered time	3.46 (1.11)	4.78 (1.40)	3.44 (1.33)	4.87 (1.41)	4.79 (1.32)	4.83 (1.41)	0.80/0.89
Self-diminishment	3.08 (1.19)	3.36 (1.55)	3.07 (1.28)	3.50 (1.74)	3.74 (1.62)	3.43 (1.65)	0.85/0.91
Connectedness	3.98 (1.19)	2.63 (1.43)	4.03 (1.21)	3.33 (1.66)	2.91 (1.45)	2.99 (1.59)	0.83/0.92
Vastness	4.07 (1.39)	3.18 (1.52)	4.08 (1.42)	3.99 (1.61)	3.49 (1.52)	3.59 (1.62)	0.87/0.90
Physical sensation	2.75 (1.21)	2.50 (1.20)	2.83 (1.46)	2.67 (1.22)	2.55 (1.23)	2.58 (1.21)	0.87/0.82
Need for accommodation	3.95 (1.21)	4.24 (1.48)	3.83 (1.26)	4.34 (1.51)	4.47 (1.41)	4.29 (1.49)	0.77/0.85
Elevation	2.90 (0.91)	2.73 (1.03)	2.97 (0.97)	2.92 (1.18)	2.94 (0.94)	2.83 (1.11)	0.87/0.91
Gratitude	3.80 (0.89)	3.42 (1.09)	3.97 (0.93)	3.54 (1.18)	3.89 (0.92)	3.48 (1.14)	0.91/0.94
Spiritual Transcendence	4.60 (1.33)	4.59 (1.45)	4.57 (1.50)	4.57 (1.56)	4.59 (1.42)	4.58 (1.51)	0.97/0.98
Anxiety	2.83 (1.42)	2.02 (1.14)	2.51 (1.36)	1.93 (1.07)	2.67 (1.39)	1.97 (1.10)	0.85/0.81
Cybersickness	-	1.77 (0.60)	-	1.57 (0.53)	-	1.67 (0.57)	- /0.92
Experience with VR	-	1.43 (1.15)	-	1.31 (1.15	-	1.37 (1.15)	-
Experience with Art	-	0.72 (0.88)	-	0.76 (0.90)	-	0.74 (0.89)	-/0.76

Large and Small refer to the scale of the art shown in VR.

Participants spent an average of 13.72 min (*SD* = 4.96) exploring the virtual environment. Time spent in the virtual environment did not significantly differ between Small (*M* = 14.19, *SD* = 4.84) and Large stimuli conditions (*M* = 13.26, *SD* = 5.08), *t*(131) = -1.08, *d* = -0.19, *p* = 0.28. On average, participants were relatively inexperienced with VR (*M* = 1.37, *SD* = 1.15) and art (*M* = 0.74, *SD* = 0.89). Unlike in Study 1, VR conditions did significantly differ in reported cybersickness, *t*(253) = -2.73, *d* = 0.34, *p* < 0.01, with participants in the Small stimuli condition (*M* = 1.77, *SD* = 0.60) reporting greater cybersickness than participants in the Large stimuli condition (*M* = 1.57, *SD* = 0.53).

Because cybersickness (only measured at Time 2) was slightly higher in the Small than Large (art) condition, and because we did find some effects of the scale of art on emotions, the correlations listed in Table 6 should be interpreted with caution. However, the same pattern of correlations between cybersickness and emotion were observed (i.e., inverse correlations between cybersickness and both awe-connectedness and elevation; positive correlations between cybersickness and anxiety, and awe-self-diminishment and awe-need for accommodation). As in Study 1, we chose to control for cybersickness in the analyses below, deviating from our preregistered analysis plan. See Supplementary material for results of analyses without controlling for cybersickness.

**TABLE 6:** Correlations between study 2 post-test variables.

Variable	1.	2.	3.	4.	5.	6.	7.	8.	9.	10.	11.	12.	13.	14.
1. Cybersickness	1	1	1	1	1	1	1	1	1	1	1	1	1	1
2. VR experience	-0.16*													
3. Art experience	-0.02	0.10												
4. Awe	0.08	-0.18**	0.00											
5. Altered time-perception	0.04	-0.14*	-0.01	0.68***										
6. Self-diminishment	0.18**	-0.13*	0.02	0.72***	0.54***									
7. Connectedness	-0.16**	-0.04	0.05	0.73***	0.33***	0.29***								
8. Vastness	-0.11	-0.12*	-0.04	0.82***	0.42***	0.40***	0.75***							
9. Physical sensation	0.12	-0.19**	-0.02	0.68***	0.29***	0.33***	0.50***	0.52***						
10. Need for accommodation	0.31***	-0.17**	0.00	0.64***	0.34***	0.45***	0.21***	0.36***	0.38***					
11. Elevation	-0.18**	-0.10	-0.00	0.39***	0.18**	0.08	0.52***	0.45***	0.37***	0.08				
12. Gratitude	-0.19**	-0.07	0.00	0.20**	0.13*	0.02	0.26***	0.19**	0.21***	0.04	0.73***			
13. Spiritual transcendence	-0.10	-0.04	-0.01	0.18**	0.16**	0.14*	0.15*	0.14*	0.01	0.17**	0.20***	0.28***		
14. Anxiety	0.44***	-0.07	-0.01	0.21***	0.13*	0.21***	0.02	0.06	0.20**	0.29***	-0.06	-0.01	-0.08	

*p < 0.05, **p < 0.01, ***p < 0.001.

#### 3.2.2 Self-transcendent emotions

To test for effects of art in VR on self-transcendent emotions, a series of 2 × 2 ANCOVAs were performed with pre- and post-VR measures of each STE as the within-subjects factor (time), VR condition (Large, Small) as the between-subjects factor, and cybersickness as a covariate. Cybersickness was a significant covariate in models predicting self-diminishment, *F*(1, 260) = 12.24, *p* < 0.01, physical sensation, *F*(1, 260) = 10.53, *p* < 0.01, and need for accommodation, *F*(1, 260) = 25.91, *p* < 0.001.

**FIGURE 6:**
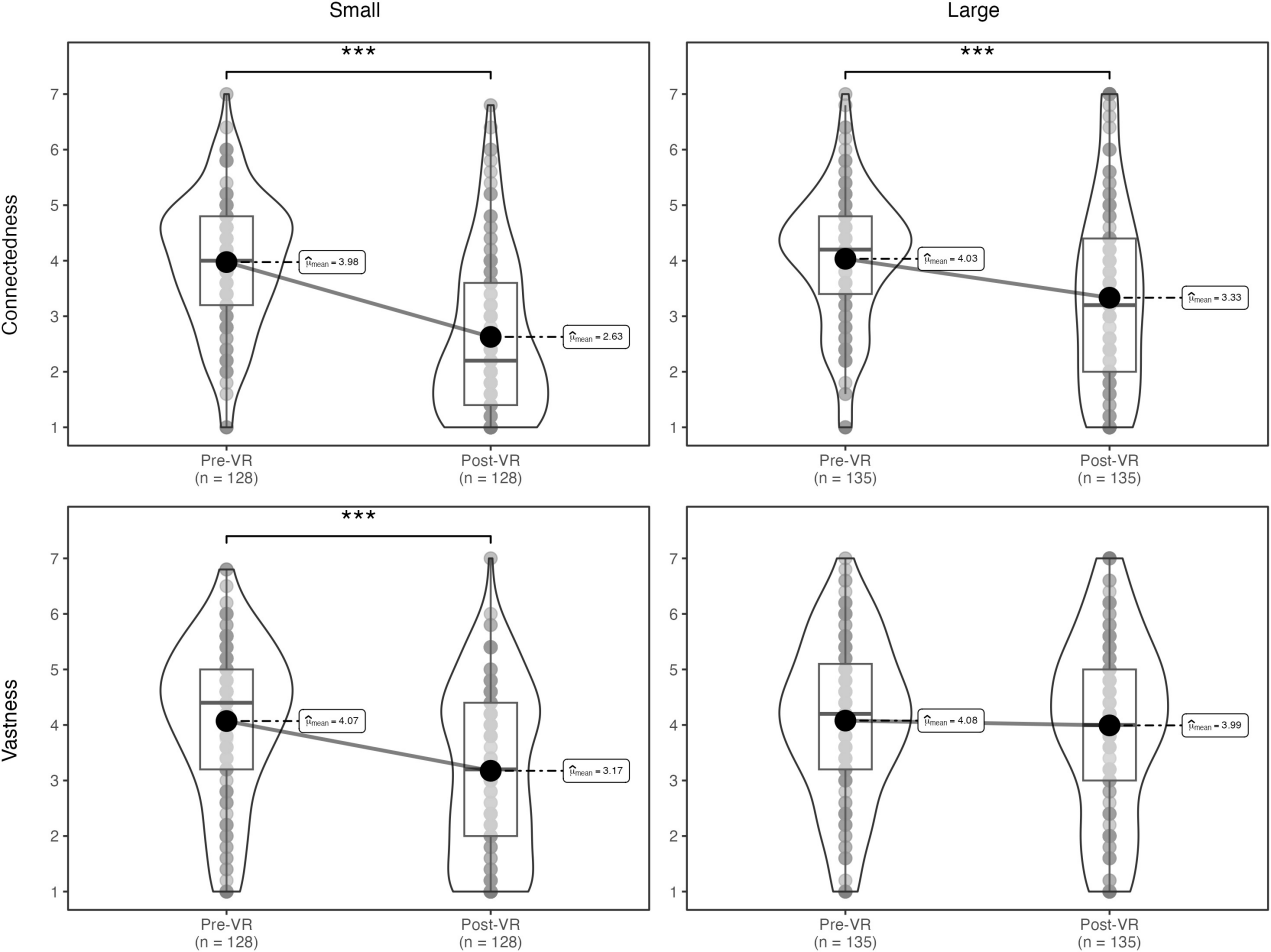
Study 2: effect of condition on dimensions of awe. ***p < 0.001.

As shown in Figure 6, after controlling for cybersickness and correcting for inflated Type I error rate, the main effect of time on awe, assessed by the full scale, was nonsignificant, *F*(1, 260) = 0.30, η_*G*_^2^ = 0.00, *p_*adj.*_* = 0.73, and there was no significant interaction with condition, *F*(1, 260) = 4.92, η_*G*_^2^ = 0.01, *p*_*adj.*_ = 0.07.

However, analyses revealed a significant main effect of time (pre- or post-test) on the altered time perception facet of awe, *F*(1, 260) = 20.75, η_*G*_^2^ = 0.03, *p*_*adj.*_ < 0.001, such that participants reported significant increases in altered time perception following the VR experience. We also found significant interactions between time and condition on state measures of the connectedness, *F*(1, 260) = 7.83, η_*G*_^2^ = 0.01, *p*_*adj.*_ < 0.05, and vastness, *F*(1, 260) = 12.18, η_*G*_^2^ = 0.02, *p*_*adj.*_ < 0.01, facets of awe. Though participants in both Small, *t*(261) = 9.45, *d* = 1.18, *p* < 0.001, and Large, *t*(261) = 5.05, *d* = 0.61, *p* < 0.001, stimuli conditions reported a decrease in connectedness from pre- to post-test, participants in the Small stimuli condition reported experiencing a much steeper decline. After viewing, participants self-indicated vastness was lower in the Small stimuli condition than before, *t*(261) = 5.88, *d* = 0.73, *p* < 0.001, whereas pre- post vastness did not differ among those in the Large stimuli condition, *t*(261) = 0.58, *d* = 0.07, *p* = 0.56.

Replicating Study 1, analyses revealed a main effect of time on state elevation, *F*(1, 260) = 6.48, η_*G*_^2^ = 0.01, *p*_*adj.*_ < 0.05, such that participants reported significant decreases in state elevation following the VR experience, but no significant interaction with condition, *F*(1, 260) = 0.42, η_*G*_^2^ = 0.00, *p*_*adj.*_ = 0.52.

Analyses revealed no main effect of time, *F*(1, 260) = 0.41, η_*G*_^2^ = 0.00, *p*_*adj.*_ = 0.52, nor any interaction between time and condition, *F*(1, 260) = 0.81, η_*G*_^2^ = 0.00, *p*_*adj.*_ = 0.48, on state gratitude.

#### 3.2.3 Spiritual transcendence

Finally, analyses revealed no main effect of time, *F*(1, 260) = 4.77, η_*G*_^2^ = 0.00, *p*_*adj.*_ = 0.08, nor any interaction between time and condition, *F*(1, 260) = 0.07, η_*G*_^2^ = 0.00, *p*_*adj.*_ = 0.79, on spiritual transcendence when controlling for cybersickness and correcting for inflated Type I error rate.

#### 3.2.4 Anxiety

**FIGURE 7:**
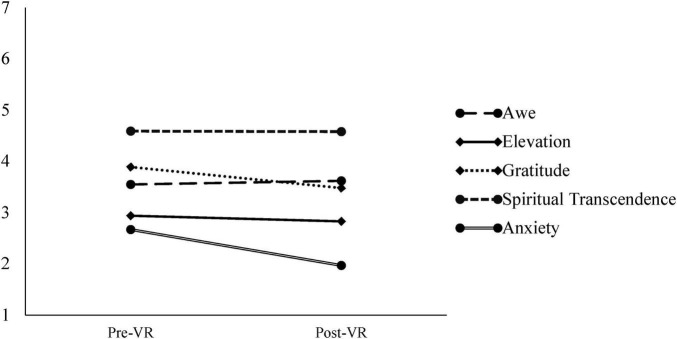
Study 2: dependent variables over time. Means are shown to illustrate change in each variable from pre-test (pre-VR) to post-test (post-VR). Variables are assessed on different scales and should not be directly compared; Awe = 1–7; Elevation and gratitude = 1–5; Spiritual transcendence = 1–6; Anxiety = 1–7.

As in Study 1, we also conducted exploratory analyses on the effect of viewing art in VR on state anxiety. Controlling for cybersickness as a covariate, *F*(1, 260) = 37.41, *p_*adj*_.* < 0.001, η_*G*_^2^ = 0.09, analyses revealed a significant main effect of time on state anxiety, *F*(1, 260) = 28.59, η_*G*_^2^ = 0.03, *p*_*adj.*_ < 0.001, such that participants reported greater anxiety before the VR experience (*M* = 2.67, *SD* = 1.39) than after (*M* = 1.97, *SD* = 1.10). A non-significant interaction between time and condition, *F*(1, 260) = 3.25, *p*_*adj*_. = 0.09, η_*G*_^2^ = 0.004, suggests that this decrease was consistent across conditions (see Figure 7).

## 4 Study 3

Contrary to our predictions, self-transcendent emotions decreased, rather than increased, from pre-test to post-test in Studies 1 and 2. In Study 3, we sought to probe two possible explanations for this unexpected finding: the presence of rather unrealistic non-playable characters (NPCs) in the virtual space and interference by cybersickness.

Although recent research suggests that awe and other positive emotions are amplified when experienced in the presence of others (Foy et al., 2025), it is possible that the presence of NPCs, which were included in the VR spaces with the intent of increasing their realism and mimicking the social exposure involved in visiting an art gallery in real life, had unintended negative effects on the elicitation of targeted emotions. Prior research suggests that facial expressions of virtual avatars are recognized with similar accuracy to facial expressions in real life (Gutiérrez-Maldonado et al., 2014), and have the ability to impact the human perceiver’s emotion (Ku et al., 2005). In our studies, the NPCs displayed motionless, neutral expressions. Although participants were not instructed to pay particular attention to the NPCs or their expressions, research demonstrating that facial expressions of virtual agents can interfere with even irrelevant perceptual tasks (Rapuano et al., 2023), suggests that the mere presence of these neutral-expressioned NPCs might have affected the emotional responses elicited by our VR intervention. Alternatively, since NPCs featured semi-realistic features and clothing but remained motionless, it is possible their presence triggered an “uncanny valley” effect (Mori et al., 2012). VR experiences with non-human characters can elicit feelings of “eeriness” if the virtual characters appear too real. Evidence from Study 1 corroborates this, as participants rated the NPCs and quite unrealistic when assessing the realism of various components of the VR environments.

In studies 1 and 2, cybersickness was a significant covariate of the effect of VR conditions on self-transcendent emotions, suggesting that some of the unexpected decreases in self-transcendent emotions could be attributed to the physical sensation of perceiving movement in the virtual space without equivalent movement in real life. Thus, in the current study, we administered a recording of the virtual environment used in Study 2 via 2D video.

### 4.1 Methods

#### 4.1.1 Participants and procedure

To investigate the effect of the presence of NPCs on the unexpected findings, we experimentally manipulated whether the NPCs were present in the virtual art gallery using a mixed repeated-measures design. Participants first completed baseline self-report measures of self-transcendent emotions and spiritual transcendence before being randomly assigned to view a 2D video recording of the “Large” condition from Study 2 with or without NPCs visible in the virtual space. After watching the video, participants completed self-report measures of self-transcendent and uncanny emotions.

Two-hundred and fifty U.S.-based adults ages 18–25 were recruited from CloudResearch Connect and compensated $3.00 to participate in this online study. This sample size was determined by a priori power calculations conducted in G*Power (version 3.1), which suggested that 234 participants were needed to have adequate power (1–β = 0.80) to detect a small to medium between-subjects effect when α = 0.05. We then oversampled by ∼10% to account for loss of data due to attrition and quality issues.

After excluding participants for completing < 50% of the survey (*n* = 4), non-U.S. or likely fraudulent IP addresses (*n* = 7), failed attention checks (*n* = 40), self-reported poor data quality (*n* = 0), a total sample of 199 participants were included in analyses (*M*_*age*_ = 22.18, 50% women, 58% college students). See Table 7 for additional sample demographics.

**TABLE 7:** Study 3 participant demographics by condition.

Demographic	NPC (*n* = 99)		No NPC (*n* = 100)		Full Sample (*n* = 199)	
	*N*	*%*	*N*	*%*	*N*	*%*
Gender						
Women	52	52	48	48	100	50
Men	45	45	49	49	94	47
Other	2	2	3	3	5	3
Race						
White	44	44	49	49	93	47
Latino	9	9	11	11	20	10
Asian	21	21	18	18	39	20
Black	19	19	16	16	35	18
Multiracial	2	2	5	5	7	4
Other	1	1	0	0	1	< 1
Religion						
Christian	41	41	36	36	77	39
Hindu	2	2	5	5	7	4
Muslim	2	2	0	0	2	1
Atheist	14	14	14	14	28	14
Agnostic	23	23	27	27	50	25
Other	1	1	5	5	6	3
Age	22.04 (2.67)		22.32 (2.20)		22.18 (2.44)	

#### 4.1.2 Measures

##### 4.1.2.1 Self-transcendent emotions and spiritual transcendence

State awe, elevation, and spiritual transcendence were assessed before and after the virtual art gallery intervention. Elevation was assessed as described in studies 1 and 2. Awe was assessed using the short-form of Yaden et al.’s (2019) awe-experience scale, validated by Graziosi et al. (2024). In contrast to studies 1 and 2, which used a trait-like measure of spiritual transcendence (STI; Abernethy and Kim, 2018), Underwood and Teresi’s (2002) state-like Daily Spiritual Experiences Scale (DSES) was used to assess spiritual transcendence in the current study. Participants were asked to indicate their agreement or disagreement with statements like, “I feel God’s presence” on a 6-point Likert-type scale (1 = strongly disagree, 6 = strongly agree).

##### 4.1.2.2 State anxiety

Anxiety was assessed as described in studies 1 and 2.

##### 4.1.2.3 Uncanny emotion

To assess the experience of uncanniness, participants completed Benjamin and Heine’s (2023) 8-item scale. Participants were asked to indicate the extent to which they agree or disagree with statements like, “I feel creeped out” on a 5-point Likert-type scale (1 = strongly disagree, 5 = strongly agree).

##### 4.1.2.4 Cybersickness

Although Study 3 employed a 2D video rather than immersive VR, cybersickness was assessed using the same measure as in studies 1 and 2 to evaluate whether the suppression of self-transcendent emotions observed in studies 1 and 2 could be attributed to VR-specific physiological discomfort. Critically, we expected the 2D presentation in the current study to elicit little to no cybersickness.

##### 4.1.2.5 Perceptions of the NPCs

The eeriness of the NPCs was assessed by Gray and Wegner’s (2012) 4-item eeriness index. Participants were asked to indicate how “frightening,” “repulsive,” “strange,” and “disturbing” they found the NPCs on a 5-point Likert-type scale (1 = not at all, 5 = an extreme amount). The verisimilitude of the NPCs was assessed by a single item assessing their perceived resemblance to humans (Ratajczyk et al., 2024) (1 = not at all, 5 = an extreme amount) and a single item assessing how realistic or unrealistic they were perceived to be (1 = extremely unrealistic, 5 = extremely realistic).

### 4.2 Results and discussion

We hypothesized that if the presence of NPCs was responsible for the suppression of self-transcendent emotions observed in studies 1 and 2, participants viewing the virtual art gallery without NPCs would demonstrate increases in self-transcendent emotions from baseline, consistent with Al-Kire et al. (2025), whereas participants viewing the virtual art gallery with NPCs would demonstrate decreases in self-transcendent emotions from baseline, consistent with results from studies 1 and 2. We also hypothesized that the effect of NPCs on self-transcendent emotion would be moderated by the perceived eeriness of the NPCs, such that participants rating the characters as more eerie would demonstrate sharper declines in self-transcendent emotion from pre-test to post-test. Additionally, we hypothesized that if the suppression of self-transcendent emotions observed in studies 1 and 2 was due to cybersickness or some other aspect specific to virtual reality, and cybersickness was reported at significantly lower levels in the current study than in studies 1 and 2, we would see a main effect of time on self-transcendent emotions, such that increases in self-transcendent emotions from baseline would be demonstrated by both experimental conditions, replicating effects observed by Al-Kire et al. (2025).

We similarly registered hypotheses about the effects of NPCs and cybersickness on state anxiety. If the pre-test to post-test decrease in state anxiety observed in studies 1 and 2 was the result of a suppression effect on emotions more generally due to the presence of NPCs, we hypothesized that state anxiety would decrease only among participants viewing the gallery in the presence of NPCs, and that the effect of the virtual gallery on state anxiety among participants in the condition without NPCs would be moderated by the extent to which the NPCs were perceived as eerie. Additionally, we hypothesized that if the change in state anxiety observed in studies 1 and 2 was due to cybersickness or some other aspect specific to virtual reality, and cybersickness was reported at significantly lower levels in the current study than in studies 1 and 2, the change in state anxiety from pre-test to post-test would be consistent across conditions.

Data were analyzed using *R* (version 4.3.3; R Core Team, 2024). Where applicable, the Benjamini-Hochberg procedure was implemented to control for inflated false discovery (Benjamini and Hochberg, 1995). Preregistered hypotheses and analytic plans can be found on OSF: https://osf.io/3f6hz/?view_only=77ca4e48ecde4e1caefab9242ddffd8c.

#### 4.2.1 Preliminary analyses

Descriptive statistics for study variables appear in Table 8 and correlations between study variables appear in Table 9.

**TABLE 8:** Descriptive statistics for study 3 variables by condition.

Variable	NPCs present (*n* = 99) M (SD)		No NPCs present (*n* = 100) M (SD)		Full sample (*n* = 199) M (SD)		α (pre/post)
	Pre-test	Post-test	Pre-test	Post-test	Pre-test	Post-test	
Awe	2.77 (1.09)	2.73 (1.08)	2.69 (1.18)	2.76 (1.15)	2.73 (1.14)	2.75 (1.12)	0.91/0.88
Altered time	2.59 (1.35)	4.09 (1.77)	2.58 (1.48)	3.72 (1.87)	2.59 (1.41)	3.90 (1.83)	
Self-diminishment	2.68 (1.47)	2.31 (1.35)	2.48 (1.38)	2.45 (1.44)	2.58 (1.43)	2.38 (1.40)	
Connectedness	3.56 (1.62)	2.49 (1.64)	3.21 (1.65)	2.58 (1.87)	3.38 (1.64)	2.54 (1.67)	
Vastness	2.80 (1.52)	2.36 (1.45)	3.05 (1.59)	2.53 (1.61)	2.92 (1.56)	2.44 (1.53)	
Physical sensation	1.96 (1.22)	1.83 (1.11)	1.91 (1.24)	2.11 (1.33)	1.93 (1.23)	1.97 (1.24)	
Need for accommodation	3.06 (1.54)	3.32 (1.81)	2.89 (1.64)	3.18 (1.53)	2.97 (1.59)	3.25 (1.70)	
Elevation	2.66 (1.42)	2.29 (1.41)	2.60 (1.27)	2.34 (1.50)	2.63 (1.35)	2.31 (1.45)	0.89/0.94
Spiritual transcendence	2.86 (1.59)	2.56 (1.57)	2.80 (1.63)	2.64 (1.65)	2.83 (1.60)	2.60 (1.61)	0.96/0.97
Anxiety	2.08 (1.26)	1.78 (1.03)	2.09 (1.31)	1.78 (1.12)	2.09 (1.28)	1.78 (1.07)	0.90/0.88
Uncanniness	-	2.25 (1.13)	-	1.96 (1.02)	-	2.10 (1.08)	-/0.95
Cybersickness	-	1.27 (0.28)	-	1.29 (0.36)	-	1.28 (0.33)	-/0.88

**TABLE 9:** Correlations between Study 3 post-test variables.

Variable	1.	2.	3.	4.	5.	6.	7.	8.	9.	10.	11.	12.	13.	14.	15.
1. Cybersickness	1	1	1	1	1	1	1	1	1	1	1	1	1	1	1
2. Uncanny	0.28***														
3. NPC eeriness	0.27**	0.64***													
4. NPC humanness	0.00	-0.21*	-0.11												
5. NPC Realness	-0.16	-0.11	-0.20	0.38***											
6. Awe	0.23***	0.10	0.07	0.15	0.46***										
7. Altered time-perception	0.21**	0.09	0.11	-0.04	0.14	0.63***									
8. Self-diminishment	0.16*	0.23***	0.26*	0.04	0.31**	0.70***	0.38***								
9. Connectedness	0.09	-0.12	-0.19	0.20*	0.40***	0.78***	0.28***	0.39***							
10. Vastness	0.08	-0.12	-0.12	0.21*	0.48***	0.80***	0.30***	0.39***	0.82***						
11. Physical sensation	0.25***	0.03	-0.04	0.20*	0.56***	0.75***	0.32***	0.38***	0.64***	0.69***					
12. Need for accommodation	0.23***	0.33***	0.25*	0.07	0.19	0.64***	0.31***	0.50***	0.28***	0.31***	0.31***				
13. Elevation	0.07	-0.31***	-0.18	0.29**	0.32**	0.51***	0.17*	0.17*	0.61***	0.62***	0.55***	0.12			
14. Spiritual Transcendence	-0.01	-0.07	-0.11	-0.00	0.18	0.30***	0.10	0.13	0.32***	0.30***	0.34***	0.15*	0.31***		
15. Anxiety	0.39***	0.49***	0.42***	0.20	0.10	0.31***	0.13	0.36***	0.11	0.16*	0.24***	0.34***	0.05	0.04	

Correlations for NPC variables (Eeriness, humanness, realness) computed using NPC condition only; *p < 0.05, ^**^p < 0.01, ^***^p < 0.001.

#### 4.2.2 Cybersickness

**FIGURE 8:**
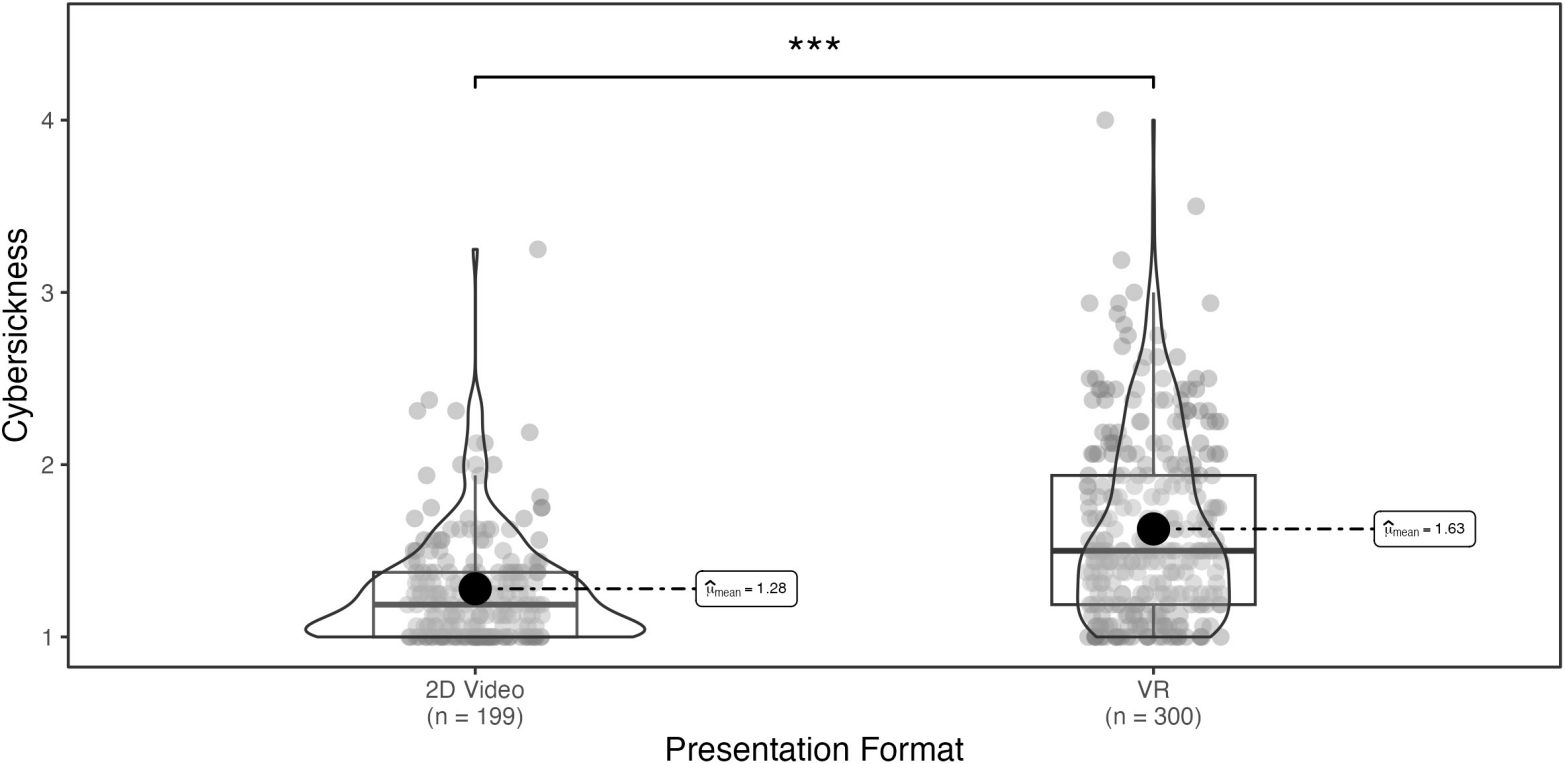
Reported cybersickness across presentation formats. ***p < 0.001.

To assess the effect of presentation medium (VR vs. 2D video) on cybersickness, we conducted an independent samples *t*-test comparing cybersickness reported in the current study with cybersickness reported in the equivalent conditions of studies 1 and 2. As the current study used the museum-like environment with large-scale art, participants in the “museum” and “large” conditions from studies 1 and 2, respectively, were collapsed to create a comparison condition. As expected, mean levels of reported cybersickness were significantly lower in the current study (M = 1.28, SD = 0.33) than observed in the equivalent conditions of the VR studies (M = 1.63, SD = 0.33), *t*(494) = -9.06, *p* < 0.001, though this comparison should be interpreted with caution given the differences between samples in recruitment method and population (see Figure 8).

#### 4.2.3 Self-transcendent emotion

To assess the effect of the virtual art gallery and presence of NPCs on self-transcendent emotions, we conducted a series of 2 × 2 ANOVAs with pre-test and post-test measures of each STE as the within-subjects factor and condition (NPCs, no NPCs) as the between-subjects factor.

Despite the significantly lower levels of cybersickness reported by participants in the current study, relative to the studies using virtual reality, self-transcendent emotions still trended in a negative direction from pre-test to post-test. Consistent with results of Study 2, the virtual art gallery had a nonsignificant effect on change in awe (assessed by the full scale), *F*(1, 197) = 0.06, η_G_^2^ = 0.00, *p*_*adj.*_ = 0.83, self-diminishment, *F*(1, 197) = 2.96, η_G_^2^ = 0.01, *p*_*adj.*_ = 0.24, need for accommodation, *F*(1, 197) = 3.80, η_G_^2^ = 0.00, *p*_*adj.*_ = 0.16, and physical sensation, *F*(1, 197) = 0.18, η_G_^2^ = 0.00, *p*_*adj.*_ = 0.67. The virtual art gallery had a significant positive effect on altered time perception, *F*(1, 197) = 98.88, η_G_^2^ = 0.14, *p*_*adj.*_ <0.001, and a significant negative effect on connectedness, *F*(1, 197) = 56.66, η_G_^2^ = 0.06, *p*_*adj.*_ < 0.001. Perceived vastness also significantly decreased from pre-test to post-test, *F*(1, 197) = 17.86, η_G_^2^ = 0.02, *p*_*adj.*_ < 0.001, replicating the effect observed in the Small condition in Study 2.

Replicating effects observed in both Studies 1 and 2, elevation significantly decreased from pre-test to post-test, *F*(1, 197) = 20.04, η_G_^2^ = 0.01, *p*_*adj.*_ < 0.001. In contrast to the VR studies, the virtual art gallery also had a significant negative effect on spiritual transcendence measured in terms of daily spiritual experiences, *F*(1, 197) = 25.62, η_G_^2^ = 0.01, *p*_*adj.*_ < 0.001.

Contrary to our hypotheses about the presence of NPCs, we found nonsignificant interaction effects between condition and time (pre-test vs. post-test), and the perceived eeriness of the NPCs did not significantly moderate the effect of NPCs on self-transcendent emotions.

#### 4.2.4 Anxiety

To assess the effect of the virtual art gallery and presence of NPCs on state anxiety, we conducted a series of 2 × 2 ANOVAs with pre-test and post-test measures of anxiety as the within-subjects factor and condition (NPCs, no NPCs) as the between-subjects factor. Replicating effects observed in Studies 1 and 2, the virtual gallery had a significant negative effect on state anxiety, *F*(1, 197) = 19.93, η_G_^2^ = 0.02, *p*_*adj.*_ < 0.001, and this effect was consistent across NPC and no-NPC conditions, *F*(1, 197) = 0.01, η_G_^2^ = 0.00, *p*_*adj.*_ = 0.97. The decrease in state anxiety from pre-test to post-test among participants in the NPC condition was not moderated by the perceived eeriness of the NPCs.

Contrary to our hypotheses, neither the lack of cybersickness nor the presence of NPCs significantly influenced the effect of the virtual art gallery on self-transcendent emotions, spiritual transcendence, or state anxiety. Instead, a pattern of decreasing emotion across time was observed across all conditions, replicating trends seen in studies 1 and 2.

## 5 General discussion

Across three studies, we investigated when and how viewing art elicits self-transcendent emotions such as awe, gratitude, and elevation. Using virtual reality to experimentally manipulate the context (Study 1) and physical scale (Study 2) of photographic art exhibits, we sought to isolate two elements theorized as necessary to self-transcendence—need for accommodation and vastness. In Study 3, we probed potential boundary conditions to clarify the perceptual and environmental factors that shape art-induced transcendence.

Contrary to our initial predictions, art viewed in VR did not reliably increase self-transcendent emotions, regardless of the context in which the art was viewed or the scale of which the art was displayed. Instead, self-transcendent emotions generally decreased or were unaffected by the intervention. However, state anxiety also consistently decreased, suggesting that the suppression effect of the intervention was not limited to positive emotions. These results were then replicated when the same virtual gallery was viewed on a computer screen (controlling for cybersickness) and when NPCs, rated as unrealistic in Study 1, were removed.

### 5.1 Implications of unexpected findings

We expected self-transcendent emotions to increase from pre-test to post-test, consistent with prior work using the same photographic art stimuli viewed on a computer screen and contrasted with non-art photos (Al-Kire et al., 2025) and a growing body of literature positioning art as a potent elicitor of positive self-transcendent emotions (e.g., Averbach and Monin, 2022; Luke, 2021). Our contradictory findings nonetheless shed light on important considerations for the study of self-transcendence.

Keltner and Haidt (2003) theorized that need for accommodation and perceived vastness are central to the experience of awe. Because these components have been confounded in prior work, we manipulated context (targeting need for accommodation) and scale (targeting vastness) independently to isolate their effects. However, this separation may partially explain our unexpected results. According to Pelowski and Akiba’s (2011) model of art perception, self-transcendence arises when viewers must revise their current schemas to make sense of the new stimulus. Consistent with this schema violation framework, Study 1 participants viewing art in a virtual warehouse—an atypical setting for an art exhibit –reported greater need for accommodation than participants viewing art in the schema-congruent setting of a virtual museum. Yet, despite eliciting a core component of awe (Keltner and Haidt, 2003), participants in the warehouse condition reported steeper decreases in self-transcendent emotions, such as gratitude, relative to those viewing art in the other contexts. If this failure to evoke transcendence reflected participants’ inability to assimilate the warehouse context with their existing schema for an art exhibit, one would also expect to observe an increase in state anxiety (Pelowski and Akiba, 2011; Taylor and Uchida, 2019). Instead, state anxiety decreased across all conditions, including the warehouse. Combined with evidence that the warehouse elicited lower perceived vastness than the church context, this pattern could support Keltner and Haidt’s (2003) hypothesis that *both* need for accommodation and vastness are necessary for awe. Further experimentation would be required to test this hypothesis specifically, but it may be that experiencing one without the other may be insufficient for self-transcendence.

A similar theoretical explanation may clarify the findings of Study 2. We hypothesized that viewing art on a large scale would heighten perceived vastness relative to viewing art on a small scale, thus increasing self-transcendence. Instead, perceived vastness decreased from pre-test to post-test in the small scale condition and remained stable in the large scale condition. Although the large condition ultimately reported greater perceived vastness at post-test than the small condition, examining change over time reveals important nuance. The decrease in perceived vastness among participants in the small condition may reflect schema incongruence. If participants entered the study with a working mental image of how art “should” appear in a gallery, encountering undersized photographic art may have violated those expectations and reduced their sense of vastness. Conversely, the large scale condition may have aligned with participants’ pre-existing schemas, eliciting no further need for accommodation or increases in perceived vastness relative to their expectations. Thus, despite greater absolute vastness, the large scale display may have failed to evoke transcendence because it lacked the necessary cognitive destabilization.

Findings from Study 3, which presented the virtual gallery in a 2D video format and varied the presence of NPCs within the virtual space seem to provide some evidence that the unexpected global decrease in self-transcendence observed in studies 1 and 2 was not solely due to the use of VR. Although cybersickness has been shown to reduce enjoyment (Shafer et al., 2019) and presence in VR (Riches et al., 2019), and emerged as a significant covariate in studies 1 and 2, substantially reducing cybersickness by opting for a 2D video format in Study 3 did not have a meaningful impact on self-transcendent emotion. Self-transcendent emotions decreased from pre-test to post-test, replicating findings from studies 1 and 2 despite markedly lower levels of cybersickness, and this pattern was consistent across conditions in which NPCs were present or absent in the virtual gallery. Although this null effect might suggest that NPCs played a negligible role in the unexpected findings of Studies 1 and 2, it is also possible that the shift from immersive VR to a 2D video format altered how the NPCs were perceived. Reduced spatial proximity and the absence of first-person embodiment may have diminished participants’ sense of shared or social space in Study 3’s 2D format, thereby limiting our ability to detect an effect.

### 5.2 Limitations and future directions

Though the decrease in self-transcendent emotions before and after viewing a virtual art gallery was replicated across three studies, additional research is needed to better understand the mechanisms of this unexpected pattern.

Taken together, the results of studies 1 and 2 suggest that stimuli may need to elicit both need for accommodation and perceived vastness to constitute awe, rather than one or the other. Future research should consider extending the current work to include conditions which challenge both schema assimilation and physical magnitude (e.g., warehouse with large scale art), and attempt to replicate our findings in older and more diverse adult populations. Participants in Studies 1 and 2 were college students who reported minimal experiences with art. It is possible that the schemas individuals have for art and art exhibits would look very different for older populations with more life experience or in more ecologically valid art settings. What conditions challenge schemas to elicit a need for accommodation, necessary for the experience of awe, may vary accordingly.

Relatedly, additional work is needed to investigate the role of *conceptual* (vs. physical) magnitude. Given the consistent decrease in self-transcendent emotions even under prototypical (i.e., museum with large-scale art) conditions with minimal cybersickness, it is possible that the stimuli used in the current research did not elicit sufficient conceptual magnitude to facilitate self-transcendence. Although the photographic artworks have been shown to elicit self-transcendent emotions in prior work (Al-Kire et al., 2025), features of their presentation in the current studies may have attenuated this effect. For instance, the randomized display of artworks, implemented to control for order effects, may have reduced curatorial coherence, thus reducing participants’ perceptions of the overall impressiveness of the exhibit. Some individual artworks or categories of artworks (i.e., nature, people, buildings, abstract) may have been perceived as more artistically complex than others or may have seemed out of place in the context in which they were displayed, contributing to a more diffuse effect on emotion. Future research should consider varying the entropy of artwork display order and collect more detailed behavioral metrics of attention to individual artworks (e.g., gaze tracking) to investigate the effects of these factors on perceived conceptual magnitude and self-transcendent emotions. Additionally, the virtual environments themselves, rated as only moderately realistic, may not have been perceived as particularly intricate or impressive, undermining the relative influence of the artwork itself on self-transcendent emotions, and perhaps resulting in a fundamentally different experience from encountering the same artwork in person. Future research should consider using technologies such as augmented reality to explore the roles of environmental verisimilitude and digital displays of art on emotional response.

Although the immersive experience of VR tends to enhance or intensify felt emotion (Markowitz and Bailenson, 2023), we observed a decrease in both positive self-transcendent emotions and state anxiety following a VR intervention using moderately positively-valenced art (Al-Kire et al., 2025). Regarding anxiety, a similar effect was found in past VR research. Playing games in VR environments resulted in overall stress reduction for university students (Shafer et al., 2023). Future work should investigate the longevity of this decreasing effect on anxiety and whether it extends to other emotions (See Silvia, 2005). Would viewing negatively-valenced art in VR suppress or intensify negative emotion? Do all emotions return to pre-test levels on the same time scale? Future VR research could also expand the lexicon of emotion words or terms used to describe aesthetic experience (cf. Schubert, 2024).

Future VR research could test mundane factors, such as sitting, standing, or even lying down (Harmon-Jones and Peterson, 2009), which might be more important for emotional experiences than we realize. Although we did not find conclusive evidence of cybersickness’ influence on self-transcendence, it is possible that small modifications to the VR intervention, such as increasing the agreement between actual and perceived movement, could reduce nausea or cybersickness (Saredakis et al., 2020), thus increasing immersion (Riches et al., 2019) and the potential for self-transcendence. Relatedly, prompting specific postures, such as leaning in, may be useful for eliciting specific positive emotions (Harmon-Jones et al., 2011), and including physiological measures could help make sense of unexpected findings when they arise.

Finally, we cannot rule out the possibility that the consistent pre- to post-test declines observed across all three studies reflect a confounding time effect resulting from the repeated-measures design. Recent work shows that self-report measures of internal states are susceptible to an initial elevation bias, in which respondents overestimate the extent to which they experience a target state upon initial assessment and report more accurately about their experience upon later reassessment (Shrout et al., 2018). Future research should replicate our design with the addition of a control condition to determine whether the observed decreases in self-transcendent emotions and anxiety are attributable to such a bias in initial assessment or to the experimental manipulation.

## 6 Conclusion

Across three studies, we used virtual reality to experimentally probe when and how art elicits self-transcendent emotions by independently manipulating need for accommodation and perceived vastness. Although viewing photographic art in VR did not reliably increase awe, gratitude, or elevation, the consistent decreases in both self-transcendent emotion and state anxiety warrant further investigation. Taken together, our findings raise the possibility that self-transcendence may require joint activation of both cognitive destabilization and magnitude, and underscores the importance of considering contextual, perceptual, and methodological factors when studying art-induced transcendence.

## Data Availability

The datasets presented in this study can be found in online repositories. This data can be found here: https://osf.io/uyj3h/overview?view_only=d60a723cf399474482e020368446563e.
