# Supplementary material for: From awe to anxiety: investigating art-induced self-transcendence using virtual reality
Source: Front Psychol. 2026 Apr 16;17:1753676. doi: 10.3389/fpsyg.2026.1753676 (PMC13130392; doi:10.3389/fpsyg.2026.1753676)
Supplement: Supplementary file 1 [file Supplementary_file_1.docx]

**Supplemental Material**

Table of Contents

[Study 1 2](#_Toc223344271)

[Additional Analyses 2](#_Toc223344272)

[Supplemental Figure 1 4](#_Toc223344273)

[Study 2 5](#_Toc223344274)

[Additional Analyses 5](#_Toc223344275)

[Supplemental Figure 2 7](#_Toc223344276)

[Study 3 8](#_Toc223344277)

[Supplemental Figure 3 8](#_Toc223344278)

[Schedule of Measures 9](#_Toc223344279)

# Study 1

## Additional Analyses

*Effect of Condition on Dimensions of Awe, without controlling for cybersickness*

| Awe: Full scale | | | | |
| --- | --- | --- | --- | --- |
|  | *DF* | *F* | *p* | *Eta squared* |
| Condition | 2,461 | 2.8371 | .06 | 0.01 |
| Awe: Altered time perception | | | | |
|  | *DF* | *F* | *p* | *Eta squared* |
| Condition | 2, 461 | 3.33 | .04 | 0.01 |
| *Posthoc* | *DF* | *t* | *p_adj._* | *d* |
| Church–Museum | 461 | 1.70 | .14 | 0.19 |
| Church–Warehouse | 461 | 2.54 | .03 | 0.29 |
| Museum–Warehouse | 461 | 0.90 | .37 | 0.10 |
| Awe: Self-diminishment | | | | |
|  | *DF* | *F* | *p* | *Eta squared* |
| Condition | 2, 461 | 2.44 | .09 | 0.01 |
| Awe: Connectedness | | | | |
|  | *DF* | *F* | *p* | *Eta squared* |
| Condition | 2, 461 | 2.29 | .10 | 0.00 |
| Awe: Vastness | | | | |
|  | *DF* | *F* | *p* | *Eta squared* |
| Condition | 2, 461 | 3.78 | .02 | 0.02 |
| *Posthoc* | *DF* | *t* | *p_adj._* | *d* |
| Church–Museum | 461 | 1.63 | .16 | 0.18 |
| Church–Warehouse | 461 | 2.74 | .02 | 0.32 |
| Museum–Warehouse | 461 | 1.18 | .24 | 0.13 |
| Awe: Physical Sensation | | | | |
|  | *DF* | *F* | *p* | *Eta squared* |
| Condition | 2, 461 | 0.25 | .78 | 0.00 |
| Awe: Need for Accommodation | | | | |
|  | *DF* | *F* | *p* | *Eta squared* |
| Condition | 2, 461 | 3.20 | .04 | 0.01 |
| *Posthoc* | *DF* | *t* | *p_adj._* | *d* |
| Church–Museum | 461 | 1.88 | .09 | 0.21 |
| Church–Warehouse | 461 | -0.49 | .63 | -0.06 |
| Museum–Warehouse | 461 | -2.39 | .05 | -0.27 |

*Effect of Condition and Time on Elevation, without controlling for cybersickness*

|  | DF | F | p | Eta squared |
| --- | --- | --- | --- | --- |
| Condition | 2,459 | 0.22 | .80 | 0.00 |
| Time | 1,459 | 10.42 | .003 | 0.01 |
| Condition*Time | 2,459 | 2.41 | .14 | 0.00 |

*Effect of Condition and Time on Gratitude, without controlling for cybersickness*

|  | DF | F | p | Eta squared |
| --- | --- | --- | --- | --- |
| Condition | 2,459 | 0.52 | .60 | 0.00 |
| Time | 1,459 | 92.91 | < .001 | 0.04 |
| Condition*Time | 2,459 | 8.26 | < .001 | 0.01 |
| *Posthoc* | DF | t | p | d |
| Church: Pre-Post | 459 | 3.85 | < .001 | 0.45 |
| Museum: Pre-Post | 460 | 4.02 | < .001 | 0.44 |
| Warehouse: Pre-Post | 459 | 8.82 | < .001 | 1.01 |

*Effect of Condition and Time on Spiritual Transcendence, without controlling for cybersickness*

|  | DF | F | p | Eta squared |
| --- | --- | --- | --- | --- |
| Condition | 2,458 | 1.03 | .36 | 0.00 |
| Time | 1,458 | 3.20 | .22 | 0.00 |
| Condition*Time | 2,458 | 1.33 | .36 | 0.00 |

*Effect of Condition and Time on Anxiety, without controlling for cybersickness*

|  | DF | F | p | Eta squared |
| --- | --- | --- | --- | --- |
| Condition | 2,459 | 0.41 | .66 | 0.00 |
| Time | 1,459 | 95.96 | < .001 | 0.06 |
| Condition*Time | 2,459 | 2.14 | .18 | 0.00 |

## Supplemental Figure 1

*Cybersickness by Condition, Study 1*


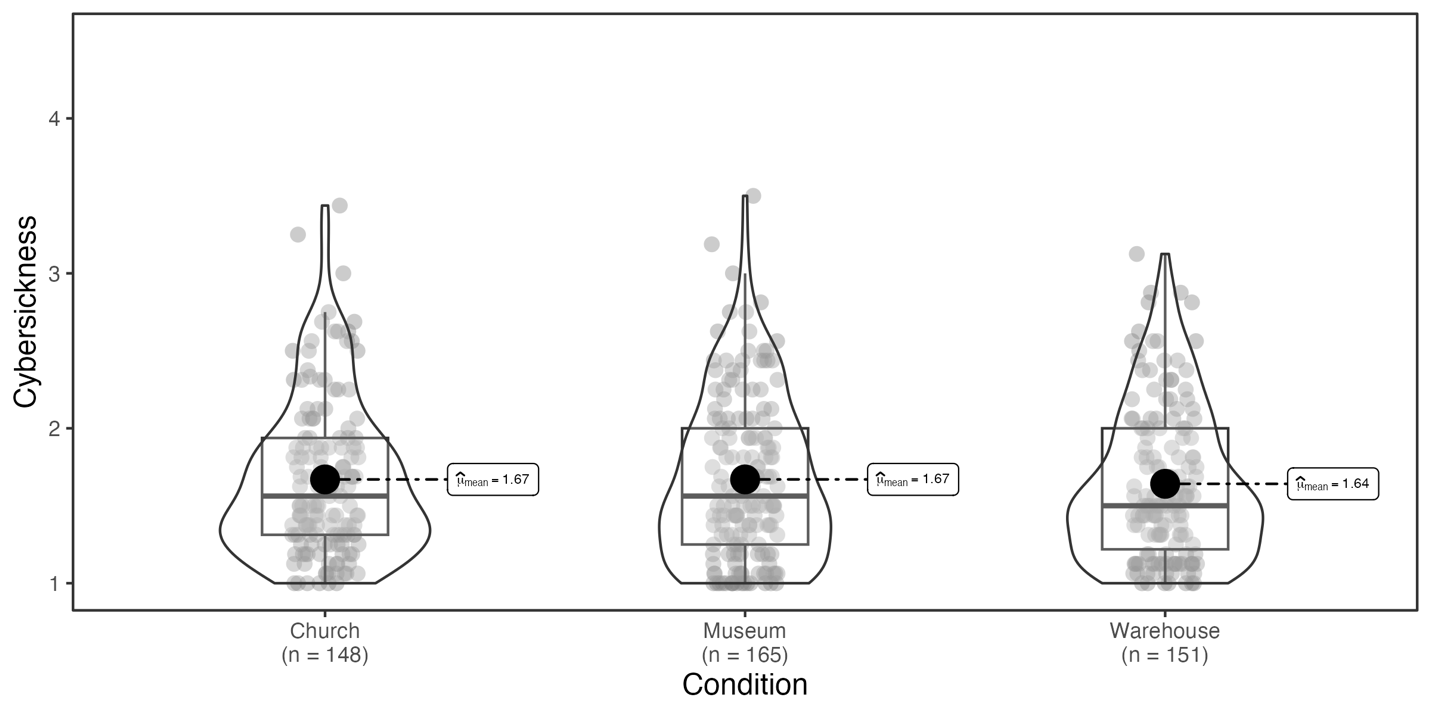


# Study 2

## Additional Analyses

*Effect of Condition on Dimensions of Awe, without controlling for cybersickness*

| Awe: Full scale | | | | |
| --- | --- | --- | --- | --- |
|  | *DF* | *F* | *p* | *Eta squared* |
| Condition | 1,261 | 2.65 | .16 | 0.01 |
| Time | 1, 261 | 0.92 | .34 | 0.00 |
| Condition*Time | 1, 261 | 5.28 | .07 | 0.01 |
| Awe: Altered time perception | | | | |
|  | *DF* | *F* | *p* | *Eta squared* |
| Condition | 1, 261 | 0.09 | .77 | 0.00 |
| Time | 1, 261 | 162.05 | < .001 | 0.22 |
| Condition*Time | 1, 261 | 0.25 | .77 | 0.00 |
| Awe: Self-diminishment | | | | |
|  | *DF* | *F* | *p* | *Eta squared* |
| Condition | 1, 261 | 0.20 | .65 | 0.00 |
| Time | 1, 261 | 9.90 | .01 | 0.02 |
| Condition*Time | 1, 261 | 0.42 | .65 | 0.65 |
| Awe: Connectedness | | | | |
|  | *DF* | *F* | *p* | *Eta squared* |
| Condition | 1, 261 | 7.53 | .01 | 0.02 |
| Time | 1, 261 | 106.02 | < .001 | 0.12 |
| Condition*Time | 1, 261 | 10.56 | .002 | 0.01 |
| *Posthoc* | *DF* | *t* | *p* | *d* |
| Small: Pre-Post | 261 | 0.45 | <.001 | 1.18 |
| Large: Pre-Post | 261 | 5.06 | <.001 | 0.61 |
| Awe: Vastness | | | | |
|  | *DF* | *F* | *p* | *Eta squared* |
| Condition | 1, 261 | 7.63 | .01 | 0.02 |
| Time | 1, 261 | 21.29 | <.001 | 0.03 |
| Condition*Time | 1, 261 | 14.49 | <.001 | 0.02 |
| *Posthoc* | *DF* | *t* | *p_adj._* | *d* |
| Small: Pre-Post | 261 | 5.88 | <.001 | 0.73 |
| Large: Pre-Post | 261 | 0.58 | .56 | 0.07 |
| Awe: Physical Sensation | | | | |
|  | *DF* | *F* | *p* | *Eta squared* |
| Condition | 1, 261 | 1.01 | .47 | 0.00 |
| Time | 1, 261 | 4.57 | .10 | 0.01 |
| Condition*Time | 1, 261 | 0.22 | .64 | 0.00 |
| Awe: Need for Accommodation | | | | |
|  | *DF* | *F* | *p* | *Eta squared* |
| Condition | 1, 261 | 0.002 | .97 | 0.00 |
| Time | 1, 261 | 15.25 | <.001 | 0.02 |
| Condition*Time | 1, 261 | 1.07 | .45 | 0.00 |

*Effect of Condition and Time on Elevation, without controlling for cybersickness*

|  | DF | F | p | Eta squared |
| --- | --- | --- | --- | --- |
| Condition | 1, 261 | 1.37 | .33 | 0.00 |
| Time | 1, 261 | 3.75 | .16 | 0.00 |
| Condition*Time | 1, 261 | 0.96 | .33 | 0.00 |

*Effect of Condition and Time on Gratitude, without controlling for cybersickness*

|  | DF | F | p | Eta squared |
| --- | --- | --- | --- | --- |
| Condition | 1, 261 | 1.57 | .32 | 0.00 |
| Time | 1, 261 | 58.24 | < .001 | 0.04 |
| Condition*Time | 1, 261 | 0.12 | .73 | 0.00 |

*Effect of Condition and Time on Spiritual Transcendence, without controlling for cybersickness*

|  | DF | F | p | Eta squared |
| --- | --- | --- | --- | --- |
| Condition | 1, 261 | 0.02 | .89 | 0.00 |
| Time | 1, 261 | 0.08 | .89 | 0.00 |
| Condition*Time | 1, 261 | 0.02 | .89 | 0.00 |

*Effect of Condition and Time on Anxiety, without controlling for cybersickness*

|  | DF | F | p | Eta squared |
| --- | --- | --- | --- | --- |
| Condition | 1, 261 | 2.50 | .17 | 0.01 |
| Time | 1, 261 | 68.23 | < .001 | 0.07 |
| Condition*Time | 1, 261 | 1.78 | .18 | 0.00 |

## Supplemental Figure 2

*Cybersickness by Condition, Study 2*


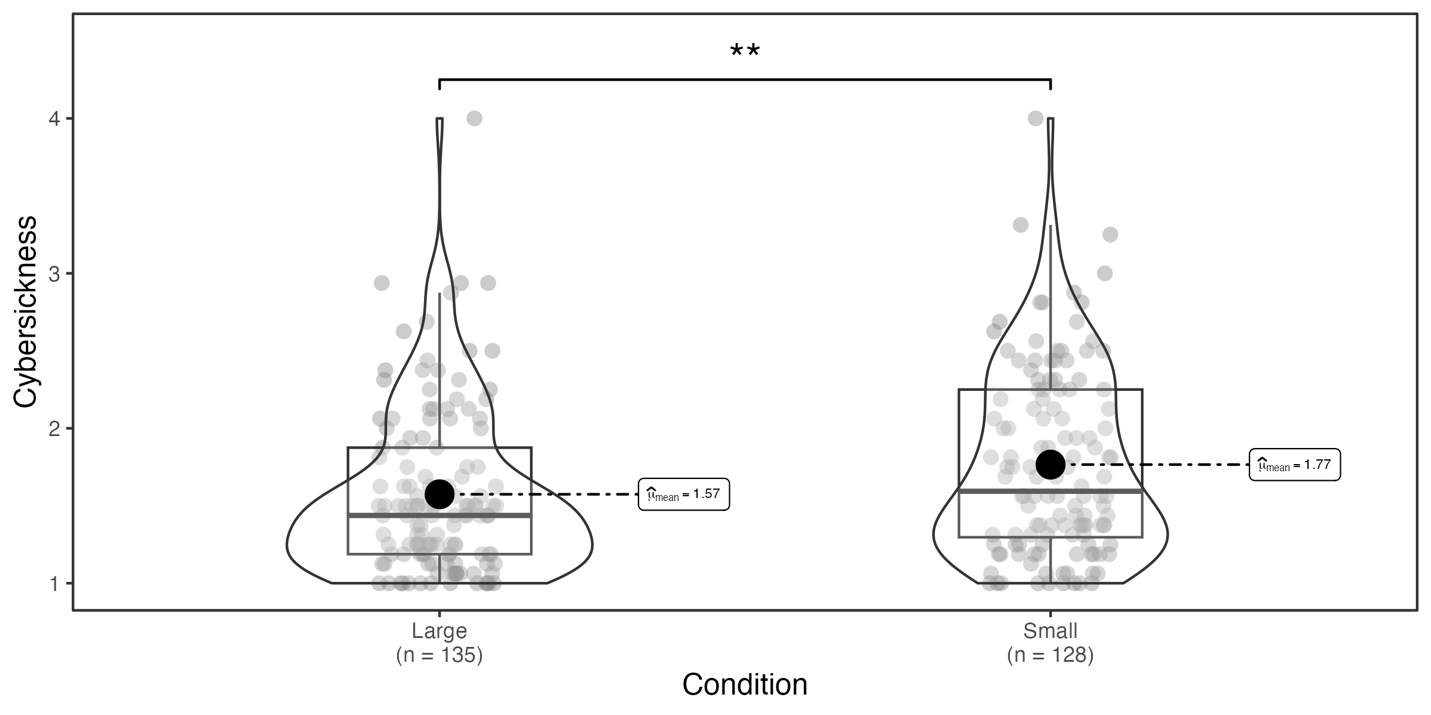


# Study 3

## Supplemental Figure 3

*Cybersickness by Condition, Study 3*


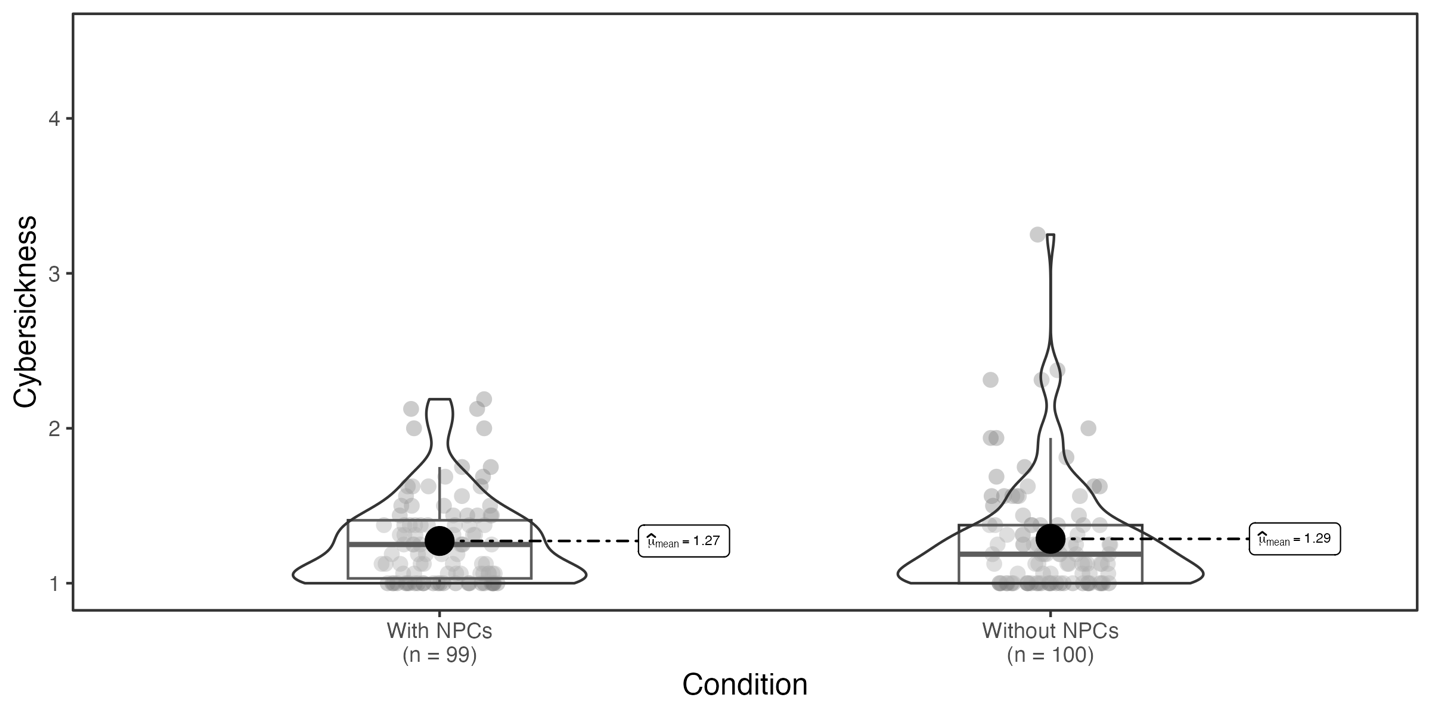


# Schedule of Measures

|  | **Study 1** | | **Study 2** | | **Study 3** | |
| --- | --- | --- | --- | --- | --- | --- |
|  | Pre-test | Post-test | Pre-test | Post-test | Pre-test | Post-test |
| **State Awe** |  |  |  |  |  |  |
| Awe-Experience Scale (Yaden et al., 2019) |  | X | X | X |  |  |
| Awe-Experience Scale, Short-form (Graziosi et al., 2024; Yaden et al., 2019) |  |  |  |  | X | X |
| **State Elevation** |  |  |  |  |  |  |
| Other-praising emotions scale (Algoe & Haidt, 2009) | X | X | X | X | X | X |
| **State Gratitude** |  |  |  |  |  |  |
| Other-praising emotions scale (Algoe & Haidt, 2009) | X | X | X | X |  |  |
| **Spiritual Transcendence** |  |  |  |  |  |  |
| Spiritual Transcendence Index (Abernethy & Kim, 2018) | X | X | X | X |  |  |
| Daily Spiritual Experiences Scale (Underwood & Teresi, 2002) |  |  |  |  | X | X |
| **State Anxiety** |  |  |  |  |  |  |
| Discrete emotions questionnaire (Harmon-Jones et al., 2016) | X | X | X | X | X | X |
| **Cybersickness** |  |  |  |  |  |  |
| Simulator Sickness Questionnaire (Kennedy et al., 1993) |  | X |  | X |  | X |

***Awe-Experience Scale (Yaden et al., 2019) / Short-form Awe-Experience Scale (Graziosi et al., 2024; Yaden et al., 2019)***

*Altered-time Perception*

1. I sensed things momentarily slow down. (SF)
2. I noticed time slowing. (SF)
3. I felt my sense of time change.
4. I experienced the passage of time differently.
5. I had the sense that a moment lasted longer than usual.

*Self-diminishment*

1. I felt that my sense of self was diminished. (SF)
2. I felt my sense of self shrink. (SF)
3. I experienced a reduced sense of self.
4. I felt my sense of self become somehow smaller.
5. I felt small compared to everything else.

*Connectedness*

1. I had the sense of being connected to everything. (SF)
2. I felt a sense of communion with all living things. (SF)
3. I experienced a sense of oneness with all things.
4. I felt closely connected to humanity.
5. I had a sense of complete connectedness.

*Vastness*

1. I felt that I was in the presence of something grand. (SF)
2. I experienced something greater than myself. (SF)
3. I felt in the presence of greatness.
4. I perceived something that is much larger than me.
5. I perceived vastness.

*Physical Sensation*

1. I felt my jaw drop. (SF)
2. I had goosebumps. (SF)
3. I gasped.
4. I had chills.
5. I felt my eyes widen.

*Need for Accommodation*

1. I felt challenged to mentally process what I was experiencing. (SF)
2. I found it hard to comprehend the experience in full. (SF)
3. I felt challenged to understand the experience.
4. I struggled to take in all that I was experiencing at once.
5. I tried to understand the magnitude of what I was experiencing.
